# Favorable epistasis in ancestral diterpene synthases promoted convergent evolution of a resin acid precursor in conifers

**DOI:** 10.1073/pnas.2510962122

**Published:** 2025-09-23

**Authors:** Andrew J. O’Donnell, Preston J. Pellatz, Caroline S. Nichols, Jonathan Gershenzon, Reuben J. Peters, Axel Schmidt

**Affiliations:** ^a^Department of Biochemistry, Max Planck Institute for Chemical Ecology, Jena D-07745, Germany; ^b^Roy J. Carver Department of Biochemistry, Biophysics and Molecular Biology, Iowa State University, Ames, IA 50011

**Keywords:** convergent evolution, epistasis, repeatability, conifers, diterpene synthases

## Abstract

A long-standing question in biology is what makes evolution more or less repeatable. Although evolution seems repeatable when observing convergent phenotypic outcomes, each episode of convergence could have been contingent on an idiosyncratic history that is not expected to repeat. Overlooking events that preceded convergent episodes therefore risks overestimating repeatability. Here, we demonstrate using resurrected ancestral proteins that the evolution of a characteristic diterpene resin acid precursor, isopimaradiene, became especially repeatable in conifers following genetic divergence that simultaneously changed the effects of phenotype-altering substitutions in diterpene synthase enzymes. We found that millions of years of epistatic change prior to three genus-level episodes of convergent isopimaradiene formation led to ancestral diterpene synthases in the family Pinaceae that served as suitable starting points for evolving identical products. However, enzymes predating the Pinaceae formed different diterpenes when the same substitutions were introduced as mutations, indicating that critical alterations in the effects of substitution occurred after the origin of this plant family. Because these alterations occurred prior to the evolution of isopimaradiene, the convergence appears dependent on a history that is less likely to repeat. Our work shows how contingency can lead to repeatable evolution of a trait in related present-day plant lineages.

## Significance

The repeated evolution of a trait through different mutations in different species is called convergent evolution, but the causes of such repeatability are rarely understood. As an example of convergence, conifers from the related genera *Picea*, *Abies*, and *Pinus* each evolved pairs of enzymes that form identical products known as diterpenes. Using reconstructed ancestral enzymes that predate the convergence, we found that several alternative amino acid replacements in recent ancestors were responsible for driving identical gains in diterpene formation. However, analysis of these replacements in more ancient enzymes showed that there was a point in conifer history when diterpene evolution became especially repeatable. We found that a genetic mechanism, termed “epistasis,” led to this enhanced repeatability.

Whether evolution is repeatable given sufficient selection pressure or contingent on prior events is a long-standing question in biology (1) and the answer is rarely straightforward. For example, several species of *Anolis* lizards have independently acquired sustained underwater rebreathing, suggesting that convergence of this trait is facile, but this repeatability is presumably dependent on shared structures that evolved beforehand (2). Similarly, at the protein level, one mechanism for such historical contingency is the presence of amino acids at particular residues (sites) that change the effects of later substitutions at nearby sites (3). Such nonadditivity is a form of epistasis and leads to contingency in evolution when the participating sites control access to a novel phenotype (3, 4). Cases of convergence among related species provide an opportunity to reconcile repeatability and contingency because they could indicate that prior changes to the epistatic disposition (or genetic “background”) of a gene in a recent common ancestor provided especially favorable starting points for repeated phenotypic transitions. Insight into how the past shaped the present is rare, however, and epistasis as a facilitator of repeatability has received little attention. Yet with a phylogeny of functionally diverse genes (or proteins) and methods of reconstructing ancestral sequences, epistasis can be detected and evaluated alongside observed evolutionary patterns (5).

**Fig. 1.**
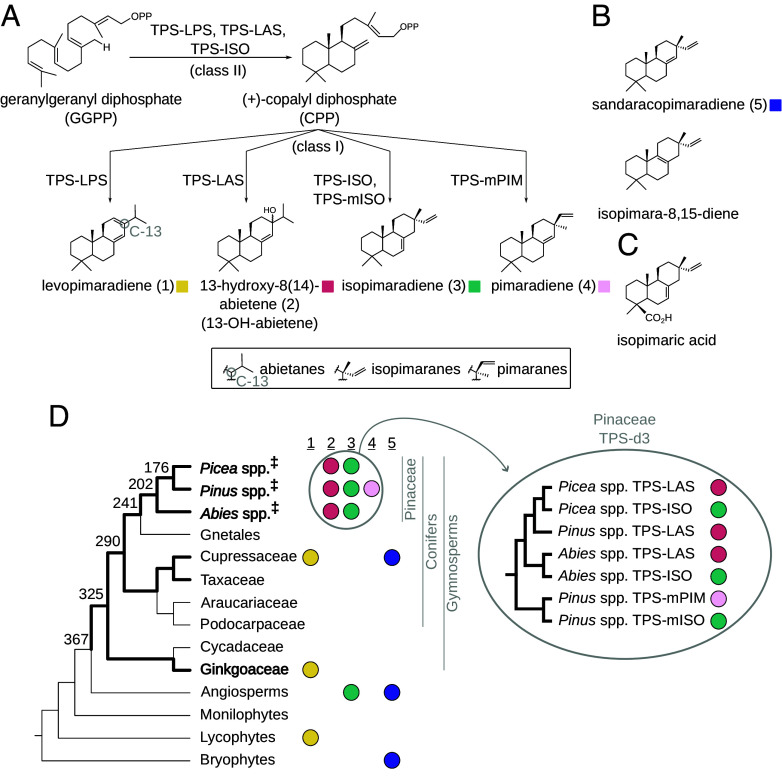
Specialized diterpene biosynthesis and convergent patterns in the TPS-d3 subfamily. (*A*) Diterpene biosynthesis is performed by TPS enzymes (arrows). Diterpenes in gymnosperms are synthesized via two sequential catalytic steps, referred to in order of reaction as “class-II” and “class-I” reactions. The final TPS products, diterpene olefins, are classified as the abietanes, isopimaranes, and pimaranes and differ structurally at C-13 (circle; see legend at the *Bottom* of panel). (*B*) Additional major products of enzymes in this study. (*C*) Diterpene olefins are the direct precursors to the diterpene resin acids (e.g., isopimaric acid) in the conifer family Pinaceae. (*D*) Phylogeny of land plants (*Left*) with estimated divergence dates (millions of years ago) shown at selected tree nodes. Bold branches indicate lineages with reported TPS-d3 enzymes capable of at least one catalytic step shown in (*A*). Lineages with bold names form diterpene olefins using TPS-d3 enzymes. "‡" indicates lineages known to accumulate mixtures of diterpene resin acids. Although many plants produce the diterpenes shown in (*A*) (*Middle*; colored circles arranged by major product), paralogous abietane-forming and isopimaradiene-forming TPS-d3 enzymes arose at the genus-level (*Right*, encircled) and are characteristic of the Pinaceae. Most characterized TPS-d3 enzymes from the Pinaceae perform both class-II and class-I reactions sequentially, but TPS-mISO and TPS-mPIM enzymes from *Pinus* perform only the class-I reactions.

Diterpenes in plants are diverse 20-carbon compounds synthesized from the precursor geranylgeranyl diphosphate (GGPP; Fig. 1*A*). While some have roles in central metabolism, most are precursors to specialized defense metabolites narrowly distributed among taxa (6–9). Diterpenes are formed by diterpene synthases (TPSs) that produce a large variety of structures, leading to diverse end products in various plant lineages. In the ecologically and commercially valuable conifer family Pinaceae, TPSs from the gymnosperm-specific TPS-d3 subfamily form tricyclic diterpene olefins that are classified as abietanes, isopimaranes, and pimaranes (Fig. 1 *A* and *D*) (10–17). Diterpene olefins are the direct precursors to the diterpene resin acids in the Pinaceae (see example in Fig. 1*C*), which are major components of bark and needle resins (18). These hallmark chemical mixtures accumulate in resin ducts and provide physical and chemical barriers against attacking insects and pathogens (18, 19).

The abietanes in the genera *Picea*, *Pinus,* and *Abies* are produced by orthologous TPSs that form primarily 13-hydroxy-8(14)-abietene (13-OH-abietene) (11–17, 20) (Fig. 1*A*). Intriguingly, TPSs that form isopimaradiene (an isopimarane) in *Picea* are most closely related to TPSs that produce abietanes in *Picea* rather than to other isopimaradiene-forming enzymes (13, 14) (Fig. 1*D*). Likewise, in *Abies*, the characterized isopimaradiene-forming TPS is most closely related to abietane-forming TPSs from *Abies* (15). Surprisingly, isopimaradiene in *Pinus* is formed via divergent TPSs that are instead most closely related to pimarane-forming enzymes from *Pinus* (12). Taxonomic, rather than functional, clustering of diverse TPSs is not uncommon (7). For example, TPSs capable of synthesizing levopimaradiene are present in *Ginkgo biloba* (e.g., levopimaradiene synthase, or “TPS-LPS” in Fig. 1*A*) and in *Taiwania cryptomerioides* (21, 22). However, the levopimaradiene-producing TPS from *T. cryptomerioides* is more closely related to other TPSs from *T. cryptomerioides* that utilize different diterpene substrates and form distinct products than to the *G. biloba* TPS-LPS. On the other hand, different pairs of abietane-forming and isopimaradiene-forming enzymes appear to have evolved repeatedly in the Pinaceae alone following gene duplication at the genus-level, suggesting that there might have been a point in conifer history at which the evolution of certain products became especially repeatable.

Given this narrow distribution of convergence among related enzymes in a single plant family, and since closely related proteins could also share similar genetic backgrounds (23), the question arises whether favorable epistasis in recent ancestral proteins can explain characteristically repeatable evolution in the Pinaceae. Because epistasis has been shown to impact access to novel products in other TPSs (24, 25), we hypothesized that TPS-d3 enzymes arrived at genetic backgrounds that had especially high potential for transitions toward isopimaradiene formation after the separation of the Pinaceae from other plants. This predicts that substitutions which drove the repeated genus-level evolution of specialized isopimaradiene formation would not have been as effective in enzymes predating this plant family. Alternatively, the effects of substitution could have been equivalent throughout TPS-d3 history, implying a diminished role for epistasis.

A powerful method of reversing the epistatic clock to test these predictions is ancestral sequence reconstruction (5). Using this approach, we first traced the functional evolution of 10 ancestral TPS-d3 enzymes from a pregymnosperm ancestor to their Pinaceae descendants to establish phenotypic histories. We next determined whether the effects of substitution were contingent on genetic history by introducing key function-changing residues as mutations to ancestral enzymes throughout the phylogeny. We found that abietanes characteristic of conifer resins have been maintained for at least ca. 325 My, whereas isopimaradiene evolved repeatedly no earlier than ca. 202-176 Mya following critical alterations to TPSs that happened to change the effects of substitution. Because these alterations occurred alongside the evolution or maintenance of other products after the origin of the Pinaceae, the convergent outcomes in this lineage appear dependent on a less repeatable past.

## Results

### The TPS-d3 Subfamily Arose from Abietane-Forming Enzymes Prior to the Separation of Extant Seed Plants.

**Fig. 2.**
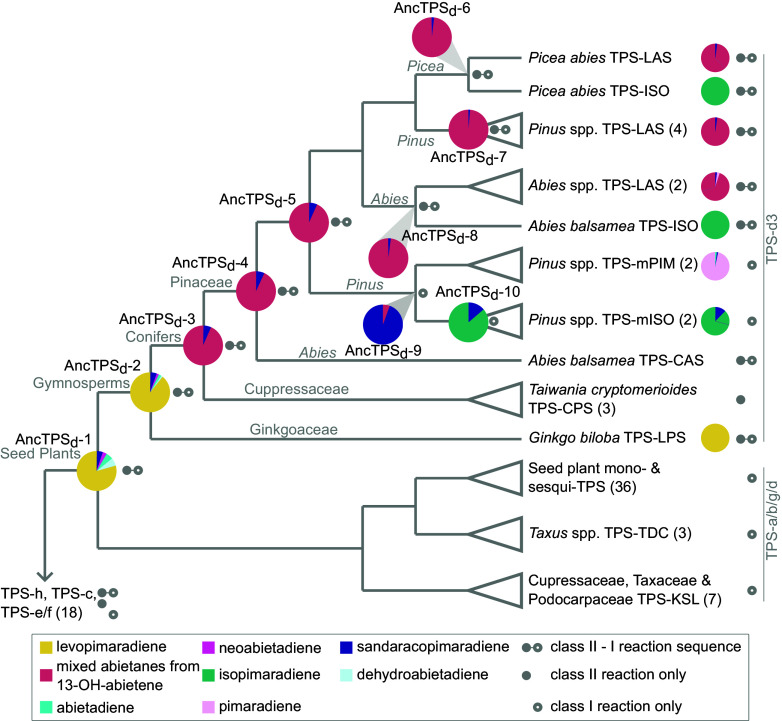
Functional origins of the TPS-d3 clade and the repeated evolution of isopimaradiene formation in different Pinaceae genera. The TPS phylogeny used for ancestral sequence reconstruction is shown. Gray branch labels indicate taxa represented in the clade to the *Right*. Pie charts at tree nodes show the relative product abundances of ancestral TPSs. The legend (*Bottom*) contains the color scheme for products and shows reactions performed by enzymes. Abietanes produced by enzymes determined to have TPS-LAS-like product profiles are assumed to be from rearrangement of 13-OH-abietene. Relative abundances of extant TPS-d3 enzymes that produce diterpene olefins in Fig. 1*A* were adapted from the literature (see main text for references). The numbers of sequences included in collapsed clades are shown in parentheses. Other TPS subfamilies from land plants included in the analysis are TPS-h, TPS-c, TPS-e/f, and the “TPS-a/b/g/d” clades, which contains multiple TPS subfamilies from seed plants.

To estimate the origin of the TPS-d3 subfamily and to distinguish between nodes preceding either duplication or speciation events, phylogenetic analysis of ca. 1,600 TPS sequences mined from a wide distribution of land plants was performed first (*SI Appendix*, Fig. S1). To generate protein sequence histories throughout the TPS-d3 subfamily, we used a reduced phylogeny of 82 representative TPSs to estimate the amino acid identities of 10 ancestral enzymes between the TPS-d3 origin and those just prior to the three genus-level duplications in the Pinaceae (Fig. 2 and *SI Appendix*, Fig. S2). The ancestral enzymes, which we named AncTPS_d_-1 through AncTPS_d_-10, are concurrent with multiple speciation and duplication nodes throughout the TPS-d3 portion of the phylogeny. Posterior probabilities of the ancestral enzymes ranged on average between approximately 0.9 and 1.0 (*SI Appendix*, Fig. S3). We therefore estimated alternative variants from seven nodes to account for sequence uncertainty (*SI Appendix*, Fig. S4; *Materials and Methods*). Phylogenetic uncertainty led to variable placement of another TPS-d clade containing enzymes from the conifer family Cupressaceae that catalyze the formation of different diterpenes and were named “kaurene-synthase-like” (22) as well as related enzymes from the Taxaceae (26) and Podocarpaceae families (*SI Appendix*, Fig. S2 and *Supplemental Methods*). We therefore estimated three ancestors from an alternative phylogeny to account for this uncertainty around the TPS-d3 origin (*SI Appendix*, Figs. S2 and S4). In either case, we inferred the TPS-d3 clade to have arisen from gene duplication at some point prior to the most recent common ancestor of extant gymnosperms over 325 Mya.

We next established the biochemical characteristics of the ancestral enzymes by expressing each in *Escherichia coli*, assaying with substrate and analyzing extracts containing the resulting products using gas chromatography–mass spectrometry (GC-MS). The diterpenes produced by TPS-d3s generally require two catalytic steps, with the first step (referred to as a class-II reaction) typically converting GGPP to the bicyclic intermediate (+)-copalyl diphosphate (CPP) (10) (Fig. 1*A*). The second step, which is the focus of this study, requires so-called class-I reactions that convert CPP to the final tricyclic diterpenes. Since most characterized TPS-d3s catalyze both reaction steps of diterpene formation sequentially in separate active sites (27, 28), we supplied GGPP as substrate unless otherwise specified.

AncTPS_d_-1, which must have been present in a common ancestor of extant seed plants over 367 Mya, ultimately gave rise to both the TPS-d3 and the TPS-a/b/g/d clades (Fig. 2), the latter of which catalyze the formation of smaller classes of specialized terpenes in angiosperms and gymnosperms and other diterpenes in gymnosperms (8). AncTPS_d_-1 converted GGPP to 80% levopimaradiene (an abietane) and to minor amounts of several other abietanes (Fig. 2 and *SI Appendix*, Table S1 contains individual product abundances for all enzymes in this study and *SI Appendix*, Fig. S5 provides representative chromatograms of ancestral enzyme assays). AncTPS_d_-2, the inferred progenitor of the TPS-d3 clade, retained production of primarily levopimaradiene—an activity that resembles the extant *Ginkgo biloba* levopimaradiene synthase (TPS-LPS) involved in the biosynthesis of ginkgolides (21). These ancestral enzymes established that the TPS-d3 subfamily arose from ancient TPSs that already specialized on the formation of an abietane (via class-II—class-I reaction sequences) prior to the separation of extant gymnosperm families.

### Isopimaradiene Formation Evolved Repeatedly from Different Ancestral Enzymes.

TPSs in modern-day Pinaceae species no longer produce levopimaradiene. Instead, TPS-d3s in this plant family catalyze the formation of either the abietane 13-OH-abietene (specifically a pair of abietane alcohols epimeric at C-13; Fig. 1*A*) and are referred to as “TPS-LASs”, or isopimaradiene and are termed “TPS-ISOs” (or “TPS-mISOs” in the genus *Pinus*, as will be discussed later), with a few producing other diterpenes (11–17, 20). To establish how AncTPS_d_-2 later gave rise to the extant sets of TPS-LASs and TPS-ISOs in present-day *Abies* and *Picea* genera, we assayed ancestral enzymes at additional nodes in the TPS-d3 phylogeny (Fig. 2). AncTPS_d_-3 arose following the split of conifers from other gymnosperms, which coincided with a major shift in enzyme products. Extracts of AncTPS_d_-3 assayed with GGPP contained a mixture of four abietane olefins in ratios similar to those found in the reported TPS-LAS from *Picea abies* (14), which we also cloned from bark tissue and assayed as a reference (*SI Appendix*, Table S1). As this profile has been reported to result from thermal degradation of the 13-OH-abietenes (20) (*SI Appendix*, Fig. S5), we lowered the GC injection temperature and analyzed extracts of all assays in the single ion monitoring mode. This revealed two peaks of the diagnostic ion *m/z* = 247 [M – C_3_H_7_]^+^ in AncTPS_d_-3, confirming formation of both reported epimers (*SI Appendix*, Fig. S5). The formation of 13-OH-abietene from GGPP persisted through multiple speciation events in conifers that led to *Abies* and *Picea* genera (as well as the *Pinus* genus), as AncTPS_d_-4 through AncTPS_d_-8 all produced 13-OH-abietene (Fig. 2). Since AncTPS_d_-6 in *Picea* and AncTPS_d_-8 in *Abies* each duplicated to yield an extant isopimaradiene-forming (TPS-ISO) and an extant abietane-forming (TPS-LAS) paralog, convergent replacements of ancestral abietane-forming activity with novel isopimaradiene formation must have occurred at the genus-level on at least two separate occasions. AncTPS_d_-7 in *Pinus*, on the other hand, is ancestral to only abietane-forming enzymes (Fig. 2).

The *Pinus* lineage is reported to have extant isopimaradiene-forming and pimaradiene-forming enzymes (12). These TPS-d3s perform just the second (class-I) step of diterpene olefin formation and are therefore considered to be “monofunctional”, while their nomenclature indicates the monofunctional formation of either isopimaradiene or pimaradiene (“TPS-mISO” and “TPS-mPIM”, respectively) (Fig. 1 *A* and *D*). Extant TPS-mISOs and TPS-mPIMs are paralogs of each other (12) and originated from duplication of an ancestral TPS that we designated as “AncTPS_d_-9” (Fig. 2). AncTPS_d_-9 did not convert GGPP to any detectable diterpene olefin (*SI Appendix*, Fig. S5), similar to its extant descendants. We therefore generated CPP as a substrate for assays with AncTPS_d_-9 by converting AncTPS_d_-8 to a construct that only forms CPP (28) (*Materials and Methods*). When this engineered protein was coupled to AncTPS_d_-9 in an assay mixture, AncTPS_d_-9 surprisingly converted the CPP to nearly 100% sandaracopimaradiene (Fig. 2), which is not a primary product of any known TPS from the Pinaceae. The next ancestral enzyme, AncTPS_d_-10, was selected to represent the extant isopimaradiene-forming TPS-d3s in *Pinus.* This enzyme shifted its product spectrum dramatically by producing largely isopimaradiene, closely resembling the present-day TPS-mISOs (12). Importantly, no traces of isopimaradiene were found in any ancestral TPS, except for about 0.1% in AncTPS_d_-6, and approximately 0.7% and 0.4% in alternative reconstructions of AncTPS_d_-3 and AncTPS_d_-9, respectively (*SI Appendix*, Table S1), revealing a clear pattern of convergence on isopimaradiene formation in three genera of the Pinaceae from ancestral enzymes with different underlying gene histories and catalytic properties.

### Repeated Evolution of Isopimaradiene Formation Occurred through Different Substitutions.

**Fig. 3.**
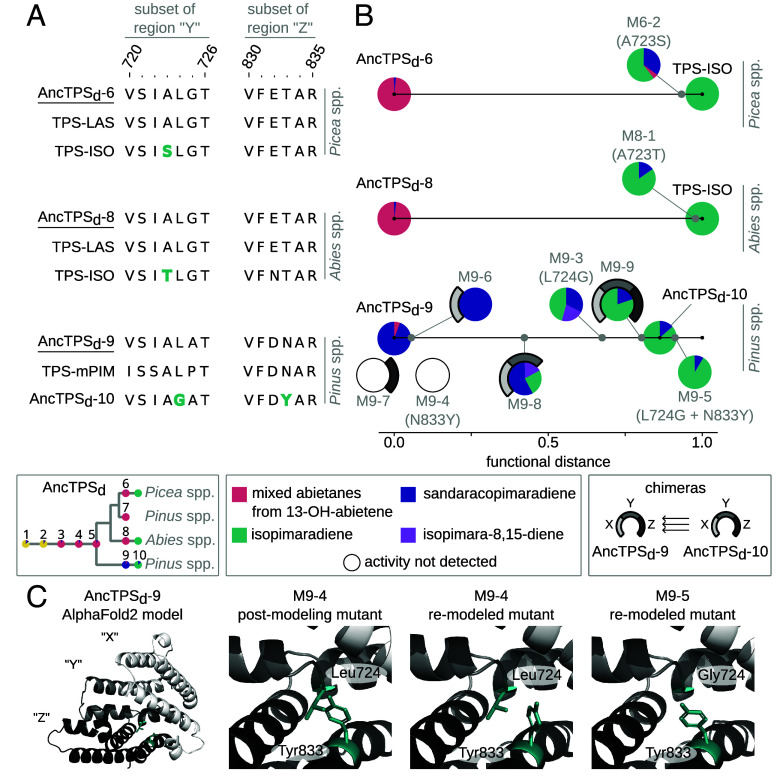
Repeated evolution of isopimaradiene formation occurred through different substitutions. (*A*) Partial amino acid alignments of selected ancestral terpene synthases (underlined) and the descendant enzymes following genus-level duplications. Site numbers for subsets of regions “Y” and “Z” correspond to full-length *Abies grandis* TPS-LAS (28). Sites affecting isopimaradiene production are shown in light green. (*B*) Functional distances (normalized Bray–Curtis dissimilarity scores) of mutants from each ancestral enzyme are shown along a horizontal axis as gray points. Distance increases for a mutant that produces any product in ratios different than its ancestor. Pie charts show products produced by each enzyme. Substitutions are indicated in parentheses and chimera configurations are indicated with shaded arcs around pie charts. The legends (*Bottom* of panel) contain a simplified TPS-d3 phylogeny, product key, and identities of chimeric regions. Enzymes shown as empty circles had impaired class-I activity. (*C*) AlphaFold2 model of regions “X” (light gray), Y (gray), and Z (black) from AncTPS_d_-9 (*Left*). An initial model for mutant M9-4 was generated by introducing N833Y to the existing AncTPS_d_-9 model in silico (“postmodeling mutant”). A new M9-4 model and an M9-5 model were generated by first introducing the N833Y substitution, and the L724G and N833Y combined substitutions, to the raw AncTPS_d_-9 amino acid sequence and then running AlphaFold2 again (“remodeled” mutants). Sites 724 and 833 are shown as light green residues.

We next sought to identify the substitutions involved in the three cases of convergence in the trajectories between AncTPS_d_-6, AncTPS_d_-8, AncTPS_d_-9, and each of their isopimaradiene-producing descendants (Fig. 3 *A* and *B*). We designated mutants with “M” followed by the number corresponding to the ancestral enzyme that served as the background construct, and appended a number to indicate the specific mutation. Where possible, the same appended number was used to indicate the same mutation in different ancestral backgrounds. For example, “M6-1” and “M8-1” contain identical mutant amino acid residues and are mutants of AncTPS_d_-6 and AncTPS_d_-8, respectively (specifically, “-1” refers to A723T and “-2” refers to A723S). A different set of mutations was investigated in the AncTPS_d_-5 background, and these mutants therefore do not strictly follow this convention. *SI Appendix*, Table S2 contains mutant codes and affected site numbers. The abietane-producing ancestors from the *Picea* and *Abies* lineages were examined first using previous mutagenesis studies (29, 30) to guide our selection of target residues. In extant abietane-forming (TPS-LAS) enzymes from the *Picea* and *Abies* lineages, substitution of an alanine with a serine at residue 723 in the protein “alpha” domain (where conversion of CPP to tricyclic diterpenes occurs) (27, 28) has been reported to lead primarily to isopimaradiene (29, 30), although this replacement apparently did not occur historically in *Abies* (Fig. 3*A*). Because AncTPS_d_-6 and AncTPS_d_-8 had replacements of Ala723 in their extant isopimaradiene-forming descendants with serine and threonine, respectively, we expected this site to contribute to the observed convergent shifts in isopimaradiene formation. In the *Picea* lineage, substitution of Ala723 with serine (A723S) in AncTPS_d_-6 indeed resulted in effective replacement of 13-OH-abietene with mostly isopimaradiene in the product profile, consistent with effects reported previously (30) (mutant “M6-2” in Fig. 3*B*). In the *Abies* lineage, the extant isopimaradiene-forming TPS (TPS-ISO) has a threonine at site 723 instead, and this replacement (A723T) in AncTPS_d_-8 also drastically shifted the product profile to primarily isopimaradiene (M8-1).

Intriguingly, in the divergent *Pinus* lineage, site 723 is an alanine in the sandaracopimaradiene-producing AncTPS_d_-9 and in its predominantly isopimaradiene-producing descendant, AncTPS_d_-10 (Fig. 3*A*). Thus, the convergent product shift to isopimaradiene in the *Pinus* lineage must have occurred via substitutions to different sites. AncTPS_d_-9 and AncTPS_d_-10 differ at 20 positions in the alpha domain (*SI Appendix*, Table S3). Among these 20 positions, nearby site 724 changed from a leucine residue in AncTPS_d_-9 to a glycine in AncTPS_d_-10. We therefore constructed the L724G mutant of AncTPS_d_-9 (M9-3) and assayed it with CPP using the coupled assay system described above. M9-3 differed substantially from AncTPS_d_-9, having supplanted most of the ancestral sandaracopimaradiene in favor of a mixture containing 46% isopimaradiene (isopimara-7,15-diene), 33% sandaracopimaradiene (isopimara-8(14),15-diene), and 22% of another isomer, isopimara-8,15-diene (Fig. 3*B*). The 8,15-diene isomer (Fig. 1*B*) was surprising because it is only known to be synthesized by a bacterial enzyme (31) and is therefore not a known product of any descendant of AncTPS_d_-9. Thus, the L724G substitution was not sufficient to drive the convergent isopimaradiene product specificity in *Pinus* as it was historically realized.

To identify other sites controlling product formation in the AncTPS_d_-9 to AncTPS_d_-10 transition, we first divided a majority of the alpha domain into three adjacent regions surrounding the CPP binding pocket, which we named regions “X,” “Y,” and “Z” (*SI Appendix*, Fig. S6). We next created single, double, and triple chimeras of AncTPS_d_-9 in which each region was replaced with that from AncTPS_d_-10 in selected combinations (M9-6 through M9-9; *SI Appendix*, Table S2). As expected, replacing all three regions in AncTPS_d_-9 with those from AncTPS_d_-10 (M9-9) resulted in a shift in CPP cyclization to yield roughly 80% isopimaradiene and 20% sandaracopimaradiene, similar to AncTPS_d_-10 (Fig. 3*B*). However, while replacing just region X on its own (M9-6) had little impact on product distribution, replacing regions X and Y together in mutant M9-8 (region Y contains residue 724) led to a product profile similar to the single mutant containing only the L724G replacement. This indicated that region X from AncTPS_d_-10 played little role in this functional transition, and suggested that the L724G substitution in region Y must be accompanied by replacements in region Z instead to complete the convergent shift. Replacement with just region Z from AncTPS_d_-10 (M9-7), however, resulted in no detectable diterpene olefins. Since region Z did not impair diterpene formation in the M9-9 chimera, epistasis between protein regions must be causing context-dependent effects of substitution.

To confirm this supposition, we first modeled the protein structure of AncTPS_d_-9 in AlphaFold2 (*Materials and Methods*) to search for potential sources of epistasis between regions Y and Z (Fig. 3*C*). Further comparison of amino acid changes between AncTPS_d_-9 and AncTPS_d_-10 (Fig. 3*A*) suggested that a derived tyrosine at site 833 in region Z of AncTPS_d_-10 would likely clash with the ancestral Leu724 in region Y of AncTPS_d_-9 (see “M9-4 postmodeling mutant” in Fig. 3*C*). Whereas the N833Y mutant of AncTPS_d_-9 caused a loss of detectable function in assays (“M9-4” in Fig. 3*B*), combining just the L724G and N833Y replacements (M9-5) revealed two striking effects: 1) the deleterious effect of N833Y was reversed with the addition of L724G, and 2) N833Y abolished all traces of the 8,15-diene isomer caused by Gly724, shifting the product profile to about 90% isopimaradiene and 10% sandaracopimaradiene (Fig. 3*B*). Remodeling the M9-4 and M9-5 proteins in AlphaFold2 allowed us to observe how the positions of mutated residues might be affected spatially (see “remodeled” mutants in Fig. 3*C*). Interestingly, this indicated that the large ancestral Leu724 residue in the impaired M9-4 mutant repels the derived Tyr833 (which is also large) away from the active site. Conversely, the smaller glycine following the L724G substitution in the fully functional M9-5 mutant appears to accommodate an inward rotation of Tyr833, strongly indicating that physical, pairwise interaction between protein residues causes context-dependent effects of substitution with Tyr833.

### Lineage-Specific Epistasis Controlled the Effects of Substitution in Different TPS-d3 Ancestors.

**Fig. 4.**
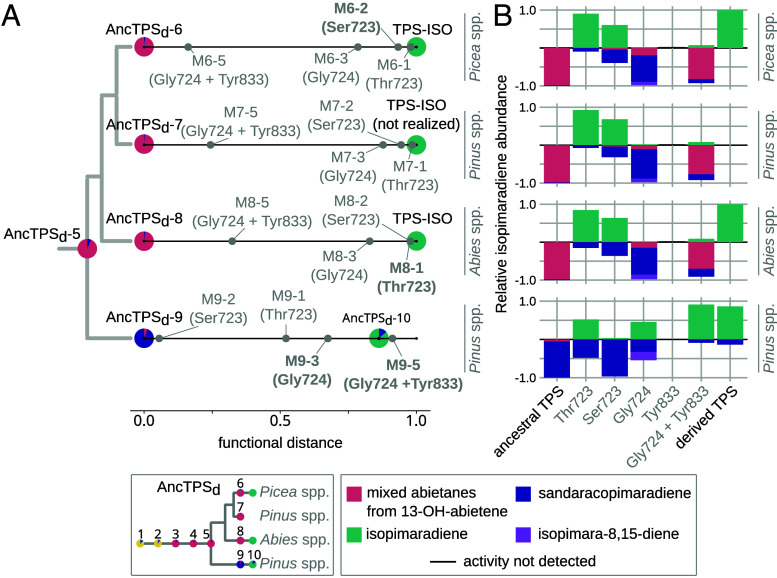
Lineage-specific epistasis controlled the effects of substitution in different TPS-d3 ancestors. (*A*) The phylogeny of AncTPS_d_-5 through AncTPS_d_-9 is shown. AncTPS_d_-6 through AncTPS_d_-8 were each mutated to contain amino acid residues that were not historically realized in any subsequent evolutionary interval (gray, parentheses). Residues that occurred historically in each interval are also shown in gray and bold for comparison. A hypothetical TPS-ISO descended from AncTPS_d_-7 is shown for comparison. Functional distances (normalized Bray–Curtis dissimilarities) of mutants from each ancestor are shown along the horizontal axis as gray points. Distance increases for a mutant that produces any product in ratios different than its ancestor. (*B*) Relative product formation of ancestors, mutants (gray), and convergently evolved (“derived”) TPSs are compared to the *Right* of each distance plot in stacked bar charts. Relative isopimaradiene abundance is shown above the *x*-axis and all other products are shown below the x-axis. The legends for both panels (*Bottom*) contain a simplified TPS-d3 phylogeny and the enzyme product key. Mixtures of abietanes produced by mutants of TPS-LAS-like enzymes are presumed to be due to residual formation of 13-OH-abietene.

We now had evidence that these repeated shifts in the product profile of TPSs in the Pinaceae occurred through different sets of substitutions and were influenced by epistasis in at least one case. We therefore sought to clarify whether epistatic effects could be more widespread across the genetic backgrounds of different TPS-d3 lineages, as this would establish whether epistasis can explain the variation in sites involved in the convergent functional transitions. To determine whether the convergent substitutions could have been utilized interchangeably for isopimaradiene formation, the Ser723 (*Picea* lineage), Thr723 (*Abies* lineage), and the Gly724 and Tyr833 (*Pinus* lineage) substitutions were compared in AncTPS_d_-6 through AncTPS_d_-9 (Fig. 4). Replacements at site 723 with either threonine or serine in AncTPS_d_-6 through AncTPS_d_-8 were comparable (mutants “M6-1” and “M6-2” through “M8-1” and “M8-2”) (Fig. 4*A*), nearly abolishing the abietane-forming functions of these enzymes in favor of approximately 60 to 90% isopimaradiene (Fig. 4*B*). In the AncTPS_d_-9 background, however, Thr723 had an intermediate effect (M9-1; Fig. 4*A*) with only ca. 50% isopimaradiene being formed (Fig. 4*B*). To our surprise, Ser723 in AncTPS_d_-9 (M9-2) produced less than 5% isopimaradiene, indicating high sensitivity of this mutation to genetic background in contrast to replacement with the chemically similar Thr723.

Among other mutations, the Gly724 replacement, which yields nearly 50% isopimaradiene when introduced to AncTPS_d_-9, drastically reduced the ancestral functions of AncTPS_d_-6 through AncTPS_d_-8 but resulted in formation of predominantly sandaracopimaradiene instead (Fig. 4*B*). While Tyr833 on its own caused loss of detectable product formation in all enzymes tested, the Gly724 and Tyr833 combination rescued the activity in every case. However, these double mutants yielded less than 10% isopimaradiene (Fig. 4*B*) and had product profiles more closely resembling those of the abietane-forming ancestors (“M6-5”, “M7-5, and “M8-5” in Fig. 4*A*). Thus, of the three convergent sets of substitutions involved in the repeated evolution of isopimaradiene formation, only one (A723T) had a consistent effect on the ancestors examined so far. Divergences in the effects of substitution therefore must have occurred following duplication of AncTPS_d_-5 such that individual TPS-d3 lineages were differentially suited toward specific replacements.

To investigate how such lineage specificity evolved, we dissected the molecular basis for the Gly724 and Tyr833 contingency on genetic background by first introducing these replacements to the more ancient AncTPS_d_-5 (“M5-3” and “M5-4”; *SI Appendix*, Fig. S7). We then replaced regions X, Y, and Z of AncTPS_d_-5 with those from AncTPS_d_-9 in all possible single, double, and triple combinations, and reintroduced the Gly724 and Tyr833 replacements to each of these chimeras. While replacements with each protein region led primarily to increased sandaracopimaradiene formation in AncTPS_d_-5, these chimeras simultaneously improved the effectiveness of Gly724 and Tyr833 in driving the convergent transition toward isopimaradiene.

### Genetic Alterations Critical for Convergence Occurred after the Origin of the Pinaceae.

**Fig. 5.**
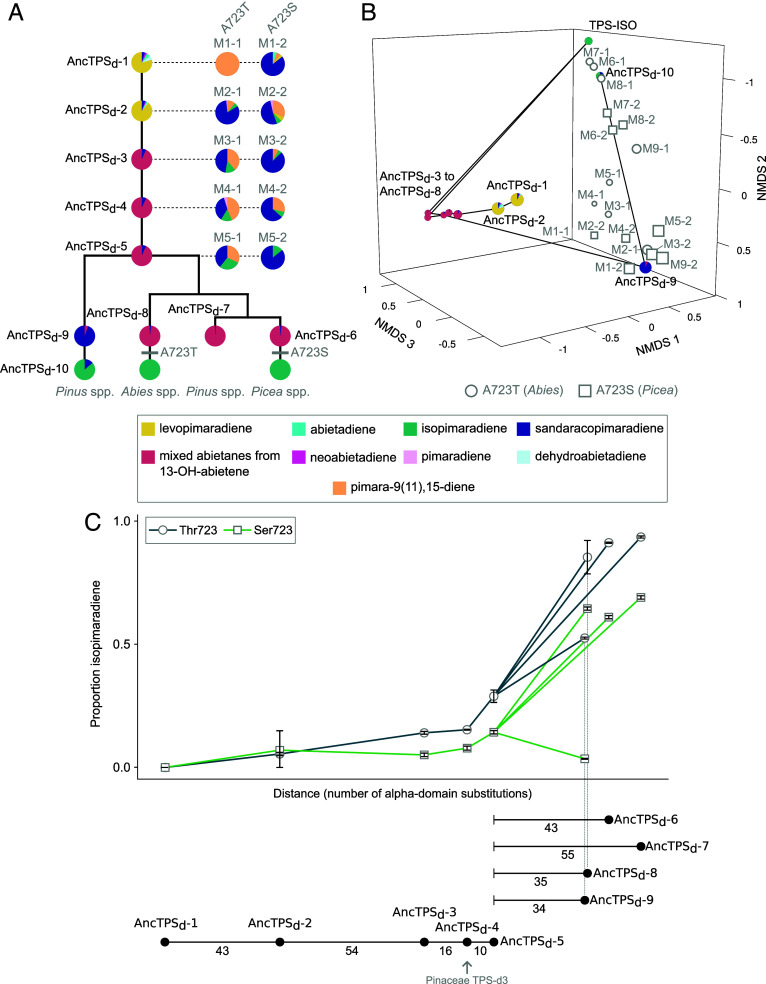
Genetic alterations critical for convergence occurred after the origin of the Pinaceae. (*A*) Relationships between AncTPS_d_-1 through AncTSP_d_-10 are indicated by the vertical phylogenetic tree. The historically realized A723T and A723S substitutions are indicated along branches with gray horizontal hatch marks. These substitutions were introduced as mutations to AncTPS_d_-1 through AncTPS_d_-5 and are shown as pie charts to the *Right* of each ancestral enzyme. (*B*) Nonmetric multidimensional scaling (NMDS) by dissimilarity score (Bray–Curtis) for all ancestral enzymes and A723S/T mutants in this study. Enzymes with similar product compositions and abundances cluster closely on all three axes. Axis units are arbitrary. The black line shows the historically traversed trajectories through this space by ancestral enzymes, and mutants are shown in gray. Mutants containing Thr723 from the *Abies* lineage (circles) and Ser723 from the *Picea* lineage (squares) are indicated. All points are scaled to position along NMDS3 and become smaller as they move toward the back of the plot. Product color scheme for panels *A* and *B* are shown in the legend (*Bottom* of panel). (*C*) The effect on relative isopimaradiene formation following replacements with Thr723 (circles) or Ser723 (squares) is tracked across ancestral enzymes by dark green and green lines, respectively. Error bars show one SD above and below means derived from at least three replicate assays. The horizontal axis is scaled to the pairwise distance (“Hamming” method; *SI Appendix*, Table S3) between each ancestor. Interval sizes are shown and the most ancient Pinaceae-derived TPS-d3 is indicated.

While our results indicated that specialization on isopimaradiene formation became accessible through substitution at sites 724 and 833 between AncTPS_d_-5 and AncTPS_d_-9 in the *Pinus* lineage, our results also showed that replacements with Thr723 and Ser723 were effective in driving convergence in *Abies* and *Picea*. Our hypothesis that TPS-d3s in the Pinaceae arrived at genetic backgrounds that were suited to the convergent transitions in function predicts that TPSs prior to the origin of the Pinaceae were unable to specialize on isopimaradiene formation through any of the historically realized phenotype-altering substitutions. To assess whether Thr723 (A723T) and Ser723 (A723S) could promote isopimaradiene production in more ancient enzymes, we introduced these replacements to AncTPS_d_-1 through AncTPS_d_-5 and examined the resulting products in the extracts from use of a previously described *E. coli* expression system that supplies TPSs with GGPP as substrate (32, 33) (mutants “M1-1” and “M1-2” through “M5-1” and “M5-2”) (Fig. 5*A*). In every case, mutation abolished most of the ancestral functions, leading primarily to elevated production of sandaracopimaradiene and an unexpected pimarane (pimara-9(11),15-diene). Levopimaradiene, abietadiene, and pimaradiene were also produced in different combinations by 4 of these mutants, each in amounts of around 6% or less of the total diterpene olefin product.

The activities of AncTPS_d_-1 through AncTPS_d_-10, all A723T and A723S mutants from this study, and a TPS producing 100% isopimaradiene (TPS-ISO), were represented in a three-dimensional “functional space” (Fig. 5*B*). We used nonmetric multidimensional scaling (NMDS) such that dissimilarities in product composition and/or abundance between enzymes were ranked (*Materials and Methods*). In the absence of epistatic divergence, the mutants containing the Thr723 and Ser723 residues should traverse nearly direct paths from any ancestral TPS toward TPS-ISO. However, this was only the case for the single mutants of AncTPS_d_-6 through AncTPS_d_-8 that we already tested (“M6” through “M8,” variants “-1” and “-2”). When these substitutions were introduced into AncTPS_d_-1 through AncTPS_d_-5, however, the resulting enzymes diffused through a different region of the space (“M1” through “M5”, variants “-1” and “-2”). This revealed that the effects of mutation at site 723 changed throughout the entirety of TPS-d3 evolution, similar in effect to a process known as “epistatic drift” (34).

Examining the effects of these Thr723 and Ser723 mutations specifically on relative isopimaradiene formation with respect to genetic distances between ancestral enzymes provided additional insight into this process (Fig. 5*C*). After a minimum of 113 alpha-domain changes between AncTPS_d_-1 and AncTPS_d_-4, there was little increase to isopimaradiene formation following mutation. However, the 10 substitutions between AncTPS_d_-4 and AncTPS_d_-5 coincided with increases in the relative ability of the Thr723 and Ser723 replacements to induce isopimaradiene formation of approximately 2- and 1.8-fold, respectively. Furthermore, we inferred AncTPS_d_-4 to be the most ancient Pinaceae-derived TPS in our study (*SI Appendix*, Fig. S1). Indeed, the potential for TPS-d3 enzymes to access high relative amounts of isopimaradiene through these mutations increased even further following divergence from AncTPS_d_-5 (with the exception of Ser723 in AncTPS_d_-9) before the trait evolved.

## Discussion

Conifer trees produce large quantities of diterpenes that are characteristic components of their resins (18). We provide here unequivocal evidence that the diterpene isopimaradiene evolved repeatedly in closely related conifer genera through the inheritance and subsequent recruitment of ancestral diterpene synthases of the TPS-d3 subfamily that were epistatically suited for nearly identical functional transitions. Mutagenesis of the most ancient enzymes in our study showed that specialization on the biosynthesis of this diterpene through single or double amino acid replacements was out of immediate reach until after the Pinaceae separated from other plants. As the potential for transitions to isopimaradiene formation increased prior to the convergent outcomes, including during intervals that coincided with the evolution or maintenance of other diterpenes, our results showcase how repeatability can arise as a characteristic trait in related species due to histories that are likely less repeatable.

A surprising finding in our ancestral enzymes was that the TPS-d3 subfamily has been forming the same abietane mixture for over ca. 290 My, and this inference was robust to both sequence and phylogenetic uncertainty (*SI Appendix*, Fig. S4). Interestingly, a recently described sample of fossilized resin (amber) was found to contain abietane carbon skeletons and was estimated to be approximately 320 My old (35), closely coinciding with the interval between AncTPS_d_-2 and AncTPS_d_-3 spanning from ca. 325 to 290 Mya. These findings suggest a longstanding function for the 13-OH-abietene produced by the TPS-LASs, which is converted to resin acids in the next step of diterpene biosynthesis in the Pinaceae. Resin acid mixtures rich in abietanes have been theorized to function as defense against conifer herbivores and pathogens through toxicity and by the sealing of wounds via polymerization (18). In any case, such long term maintenance of these enzymes appears to have provided phenotypically neutral avenues from AncTPS_d_-3 to AncTPS_d_-6 and AncTPS_d_-8 prior to the convergent transitions, which fortuitously brought the convergent trait within mutational reach of multiple TPS-d3 enzymes.

The persistent presence of abietane diterpenes throughout conifer history was accompanied by isopimaradiene more recently. While little is known about the specific ecological role of isopimaradiene, it could be more than coincidence that one of the deadliest insect pests of *Pinus*, *Picea,* and *Abies* are bark beetles, whose weevil (Curculionidae) ancestors arose on gymnosperms some time after ca. 228 Mya (36–38). Since these Pinaceae genera were separate lineages by about 176 Mya, the origin of bark beetles after ca. 90 Mya (36) may well have driven isopimaradiene convergence. Intriguingly, the sites utilized for the convergent shifts appeared to occur historically through substitutions at sites that maximized the relative formation of isopimaradiene (Fig. 4). This raises the possibility that epistasis localized to individual TPS-d3 lineages can influence the specific molecular forms that arise in response to an ecological challenge.

It is worth noting, however, that alternative mutational paths can lead to the same phenotypic outcome but remain unrealized (39–41). In our case, AncTPS_d_-7 from *Pinus* formed primarily isopimaradiene when the Ser723 and Thr723 replacements were introduced (Fig. 4), yet this path was evidently not traversed. Additionally, a distantly related TPS in wheat can produce isopimaradiene (42), suggesting that convergent access to this phenotype might arise unexpectedly in other plant lineages. Although the genetic backgrounds (and therefore epistatic constraints) in wheat TPSs differ from those in the TPS-d3 subfamily, repeatability across distantly related enzymes could be understood in terms of energy landscapes. TPSs that form specific products do so in large part by guiding carbocation intermediates through a series of precise rearrangements as determined by the chemistry and geometry of the enzyme active site and substrate (43). The formation of pimaranes and isopimaranes by class-I TPSs is initiated via diphosphate ionization from CPP followed by alternative stereo-specific ring closures that result in either pimarane- or isopimarane-type configurations of the C-13 methyl and vinyl groups in the final product (Fig. 1*A*) (10). The reaction is terminated via deprotonation at one of several nearby carbon atoms in the terminal intermediate, resulting in various possible double bond isomers of the final product (10). Thus, in the case of any TPS that convergently evolved isopimaradiene formation, several mechanistic “decisions” are made as the enzyme and substrate navigate energetically favorable valleys of an energy landscape (44) that ultimately lead to the formation of the same product. We assume that ancestral enzymes prior to product convergence were able to plot novel courses toward identical product wells on the energy landscape following just a few mutations, whereas the landscapes of epistatically inhibited enzymes likely contained insurmountable peaks that obstructed similar decision chains even following several rounds of substitution. Future studies of TPS functional evolution that include mutations not realized historically and consider the energetics associated with TPS functional transitions (45) are warranted to understand the full extent of repeatability and the extent to which such energy landscapes either facilitate or restrict convergence.

Epistasis is known to affect mutational paths to novel phenotypes (39) and alter the fitness of specific mutations (46). Indeed, mutational dependence on genetic background has been shown in another class of TPSs (24, 25). Our work complements these studies by demonstrating how more than 100 My of protein divergence following the origin of an enzyme subfamily can provide especially favorable starting points from which repeated trait gains can occur. That the recent convergent episodes were contingent on Pinaceae-specific genetic changes implies that some traits could either take millions of years to re-evolve in other plant lineages, or perhaps never evolve in some due to the idiosyncrasies of history.

## Materials and Methods

### Divergence Times and Phylogenetic Reconstruction of Land Plant Terpene Synthases.

Divergence times of species were estimated using the median values derived from three recent studies (47–49). To construct the diterpene synthase phylogeny (*SI Appendix*, Fig. S1) we used BLAST to retrieve TPSs from 83 gymnosperm transcriptomes from OneKP (50) and from 21 genomes across all major land plant divisions. We then performed maximum likelihood phylogenetic analysis and used the best tree for display (See *SI Appendix* for further methodological details).

### Ancestral Sequence Reconstruction.

All ancestral sequence reconstruction analyses were performed using PAML version 4.9 (51) assuming the Jones, Taylor, Thornton + gamma substitution model and the TPS phylogenies (*SI Appendix*, Fig. S2). See *SI Appendix* for detailed lists of sequences used and phylogenetic methods. “AltAll” (52) variants for AncTPS_d_-1 and AncTPS_d_-2 were synthesized and assayed, as well as “AltAlpha” variants for five additional ancestral sequences in which all residues in regions X, Y, and Z (defined in *SI Appendix*, Fig. S6) were replaced if the second-best amino acid had a probability of greater than 0.2. The phylogenetic tree used for estimation of our primary ancestral estimates (*SI Appendix*, Fig. S2) included several recently published fern and lycophyte sequences (53). We also characterized three ancestors (AncTPS_d_-1.2, -2.2, and -3.2) from another phylogeny (*SI Appendix*, Fig. S2) to account for phylogenetic uncertainty. The tree used for the estimation of these proteins was constructed prior to the publication by ref. 53 and therefore did not contain the additional fern and lycophyte sequences. All alternative ancestral enzyme activities are shown in *SI Appendix*, Fig. S4.

### Molecular Cloning and Mutagenesis.

DNA encoding each ancestral sequence was optimized for expression in *E. coli*, synthesized by Twist Bioscience, and ligated into pDEST17 using the Gateway LR Clonase II kit from Invitrogen. Our *P. abies* TPS-LAS reference was amplified from *P. abies* complementary DNA isolated from bark tissue using primers that targeted sequences encoding protein globular domains, as defined previously by Martin et al. (14), and cloned into pDEST17. Site-directed mutagenesis was carried out using overlapping primers containing desired base changes and PCR with Phusion DNA polymerase from ThermoFisher Scientific. An “AD*XX*D” protein (*SI Appendix*, Fig. S5) was constructed based on a previously reported mutant (28) using mutagenic primers that changed Asp621 to an alanine in AncTPS_d_-8 (numbered according to *Abies grandis* TPS-LAS), allowing for class-II-only formation of CPP while preventing class-I catalysis (*SI Appendix*, Fig. S5). Chimeric mutants and AltAlpha variants were constructed with in-fusion primers that targeted either inserts or vector backbones, amplified with Q5 Hot Start DNA polymerase (New England Biolabs), and ligated using Exonase II from Vazyme Biotech. *SI Appendix*, Fig. S8 shows the spatial arrangements of single- and double-site mutants and chimeric regions. We also generated a pDEST17 empty vector control by digesting pDEST17 with BsrGI restriction enzyme and religating the vector backbone. All constructs were propagated in Top10 chemically competent cells (Invitrogen) and were verified by sequencing.

### Heterologous Gene Expression and Enzyme Assays.

TPS-d3 enzymes typically possess an N-terminal “D*X*DD” motif required for the class-II protonation-initiated cyclization reaction (6–10), and this motif was found to be present in all ancestral enzymes following initial sequence estimation with the exceptions of AncTPS_d_-9, AncTPS_d_-9 AltAlpha, and AncTPS_d_-10. TPSs that perform class-I reactions have a C-terminal “DD*XX*D” motif involved in substrate coordination (6–10) (*SI Appendix*, Fig. S8), and this motif was present in all 10 estimated ancestral TPS sequences as well as in all of their alternative reconstructions (*SI Appendix*, Fig. S6). For enzyme characterization, we used the *E. coli* expression and assay system for diterpene synthases established by Martin et al. (14) with some changes. For most constructs, 100 μL suspensions of soluble *E. coli* protein containing heterologously produced TPSs in diterpene synthase assay buffer (14) were initiated in triplicate by adding 50 μM *E*,*E*,*E*-GGPP (Sigma) in 1.5 mL glass vials and incubated at 30 °C for 1 h. Coupled assays containing the AD*XX*D construct were composed of 1:1 mixtures (by volume) of each extracted enzyme and incubated as the above. Reactions were stopped by addition of 100 μL ethyl acetate containing 100 μM progesterone as internal standard and were immediately vortexed for 30 s, centrifuged for 30 s at 14,000 G at 4 °C, and the organic layer was removed for GC-MS analysis. For rapid screening of the A723S/T mutants in the AncTPS_d_-1 through AncTPS_d_-5 backgrounds, an established metabolic engineering system was used (32, 33).

### GC-MS.

1 μL of each assay extract was injected into an Agilent 6890 instrument at 270 °C with splitless transfer to an Optima 30 m × 0.25 mm fused silica capillary column (Machery-Nagel) at 7 psi and held at 40 °C for 1 min. A 7.5 °C/min ramp to 280 °C was applied and then held for 5 min. When using ion monitoring, the injection temperature was reduced to 170 °C and fragments with a mass-to-charge ratio of 247 were monitored. Peak identities were verified using authentic standards synthesized in-house, except for isopimara-8,15-diene and pimara-9(11),15-diene, which we identified through a combination of mass spectra and relative retention times of the products of isopimara-8,15-diene and pimara-9(11),15-diene synthases, respectively (31, 54).

### Data Analysis.

Relative product abundances of all enzymes were calculated using the abundance of each diterpene peak relative to the sum of total diterpenes in each GC-MS trace. A minimum of three replicates were used to generate the proportions shown in pie and bar charts. Replicate means with ± 1 SD are given in *SI Appendix*, Table S1. *SI Appendix*, Tables S4 and S5 provide abundances of class-I cyclization products calculated as the logged total diterpene olefin peak area in each trace, as well as the abundance of isopimaradiene (logged), for each construct characterized in the metabolically engineered and in vitro systems, respectively.

Functional distances were calculated by first obtaining the Bray–Curtis dissimilarity scores between each mutant (or descendant) and the ancestral TPS product abundances. To facilitate comparisons in effect magnitude of different mutations, we calculated the “functional distance” by normalizing all scores to that of an enzyme producing 100% isopimaradiene.

Our functional space in Fig. 5*B* was constructed by performing NMDS using the Bray–Curtis dissimilarity scores between all enzymes shown. Axes do not reflect absolute changes in enzyme product composition or changes in particular TPS products. Enzymes that cluster close together have similar product composition and/or relative abundances. NMDS represented the Bray–Curtis distances with a stress of 0.027.

### Protein Modeling.

Protein models were generated using AlphaFold2 (55) and the amino acid sequences of the full globular domains of ancestral sequences. Only regions X, Y, and Z are shown in Fig. 3*C*. Postmodeling mutagenesis was performed on the AlphaFold2 model in PyMol (56) with the most probable rotamers chosen.

## Supplementary Material

Appendix 01 (PDF)

## Data Availability

All study data are included in the article and/or *SI Appendix*.
