# Supplementary material for: Favorable epistasis in ancestral diterpene synthases promoted convergent evolution of a resin acid precursor in conifers
Source: Proc Natl Acad Sci U S A. 2025 Sep 23;122(39):e2510962122. doi: 10.1073/pnas.2510962122 (PMC12501191; doi:10.1073/pnas.2510962122)
Supplement: Supplementary file 1 — Appendix 01 (PDF) [file pnas.2510962122.sapp.pdf]

PNAS

Supporting Information for

Favorable epistasis in ancestral diterpene synthases promoted convergent evolution of a resin acid precursor in conifers

Andrew J. O'Donnell, Preston J. Pellatz, Caroline S. Nichols, Jonathan Gershenzon, Reuben J. Peters, Axel Schmidt

Corresponding author: Andrew J. O'Donnell
Email: aodonnell@ice.mpg.de

This PDF file includes:

Supplemental Methods
Tables S1 to S5
Figures S1 to S8
SI References

Supplemental Methods

Phylogenetic reconstruction of land plant terpene synthases

A large-scale phylogeny of terpene synthases (TPSs) from land plants was constructed using 83 gymnosperm transcriptomes (peptide translations) retrieved with the “Acrogymnospermae” tag in the “onekp” R package (1). Redundant sequences were removed from each transcriptome and all data sets were combined with the genomes (non-redundant predicted proteomes) of 3 ANA-grade angiosperms (*Amborella trichopoda* (2), *Brasenia schreberi* (3), *Nymphaea colorata* (4)), Chloranthales (*Chloranthus sessilifolius* (5)), 3 magnoliids (*Aristolochia fimbriata* (6), *Cinnamomum kanehirae* (7), and *Liriodendron tulipifera* (YP108A v1.1, DOE-JGI, http://phytozome-next.jgi.doe.gov/)), *Arabidopsis thaliana* (8), 7 gymnosperms from 5 families (*Abies alba* (9), *Cycas panzhihuaensis* (10), *Ginkgo biloba* (11), *Picea abies* (12), *Pinus taeda* (13), *Torreya grandis* (14), and *Welwitschia mirabilis* (15)), 3 bryophytes (*Anthoceros angustus* (16), *Marchantia polymorpha* (17), and *Physcomitrella patens* (18)), 1 lycophyte (*Selaginella moellendorffii* (19)) and 2 ferns (*Azolla filiculoides* (20) *and Ceratopteris richardii* (21)). We performed a local BLASTp search against this library using the previously described TPS-ISO from *P. abies* (22) and an e-value cutoff of 0.001. The resulting 3,568 sequences were passed through a length filter of 350 to 900 residues. Sequences retrieved by BLAST were combined with 103 functionally characterized TPSs, which included all characterized TPS-d3 sequences known to us. Eight sequences from *G. biloba* with masked amino acid identities were removed, resulting in a total of 1,602 sequences for phylogenetic analysis. All sequences were aligned in MAFFT (23). This alignment was analyzed in RAxML-NG (24) with 300 bootstrap replicates assuming the Jones, Taylor, Thornton (JTT) + gamma (G) model of amino acid substitution (convergence was reached with a cutoff of 0.03), and support values are shown at selected nodes of the best-scoring tree. The tree was rooted with a moss clade containing *P. patens* CPS/KS (25). The final phylogeny was visualized in the “ggtree” R package (26).

Ancestral Sequence Reconstruction

To gather representative terpene synthases for the reconstruction of our primary ancestral sequence estimates, we used the online BLAST tool from UniProt (www.uniprot.org) using default settings and the previously characterized TPS-ISO from *Picea abies* (22) and retained all functionally characterized protein sequences. Additional characterized proteins were manually identified in the literature and retrieved from Genbank and have the following accession numbers: TcTPS-CPS1, KT588482; TcTPS-CPS2, KT588483; TcTPS-CPS4, JN587309; PtTPS-PIN2, AF543527; PaTPS-PIN, AY473622; PtTPS-TERP, AF543529; AgTPS-CAM, U87910; AgTPS-MYR, U87908; PaTPS-MYR, AY473626; AgTPS-PHEL, AF139205; PaTPS-LIM, AY473624; PaTPS-FAR, AY473627; PtTPS-FAR, AF543528; PaTPS-LIN, AY473623; AgTPS-TERP, AF139206; PtTPS-PIN1, AF543530; AgTPS-PIN, AF139207; AgTPS-LIM, AF006193; PaTPS-LON, AY473625; AgTPS-HUM, U92267; AgTPS-SEL, U92266; AgTPS-BIS, AF006195; PaTPS-BIS, AY473619; PmTPS-FAR, HQ214483; GbTPS-FAR, KM248383; TcTPS-KSL5, KT588490; TcTPS-KSL6, KT588487; TcTPS-KSL7, KT588488; HsTPS-LS, OL989441; PdTPS-LS, OL989442; SmTPS-DTC3, AB898932; SmTPS-CPSKSL1, JN001323; OjTPS-MS, OL989443; OsTPS-CPS, OL989450; HpTPS-DTC1, LC128408; MpTPS-DTPS5, OL989446; PgTPS-CPS, GU045755; SmTPS-TPS9, JX413782; ZmTPS-KSL3, DAA36069; PgTPS-KS, GU045756; SmTPS-KS, AB898933; PpTPS-CPSKS, AB302933. To mitigate potential sampling bias among gymnosperms, we also used BLAST with *P. abies* TPS-ISO against all gymnosperm transcriptomes on OneKP (1) and retained only sequences that passed through a stringent length cutoff of 750 to 900 amino acids (the model diterpene synthase TPS-LAS from *Abies grandis* has a total length of 868 amino acids (27)), and all identical matches to characterized proteins already mentioned above were removed. The final set of sequences retrieved from OneKP, and the plant families represented, were: BTTS-2006591 (Taxaceae), ROWR-2004389 (Podocarpaceae), XLGK-2000274 (Podocarpaceae), and JZVE-2002778 (Podocarpaceae). These four sequences formed a clade with previously characterized sequences from Cupressaceae (28) which were termed “kaurene-synthase-like” (KSL). The more extensive phylogenetic analysis shown in Fig. S1 indicated that this clade also includes related enzymes from the Taxaceae (29) that were not included in the ancestral sequence estimations. To generate trees used for estimation of ancestral sequences, all sequences were aligned with MAFFT (23), predicted N-terminal transit peptides were removed, and the resulting alignment was analyzed in RAxML-NG (24) using the “all” option, the JTT+G substitution model, and 1,000 bootstrap analyses. The best tree was used in PAML (CODEML) (30) to generate ancestral sequences. Artificial insertions in ancestral sequences were identified using parsimony and removed manually.

Table S1. Separated relative product abundances following all assays with ancestral and mutant enzymes. Each value is the mean relative proportion of each diterpene in the total product pool (± 1 standard deviation) following enzyme assays. “n.d.”, diterpene not detected. “Pa”, *Picea abies*. *, product can result from non-enzymatic rearrangement of 13-hydroxy-8(14)-abietene (“13-OH-abietene” in the main text) (31). Palustradiene and levopimaradiene were summed due to co-retention in some assays.

Construct name	pimara-9(11),15-diene	isopimara-8,15-diene	pimaradiene	sandaracopimaradiene	isopimaradiene	palustradiene* + levopimaradiene*	unknown diterpene(s)	dehydroabietadiene	abietadiene*	neoabietadiene*
AncTPSd-1	n.d.	n.d.	n.d.	0.05 ± 7.8 x 10^{-3}	n.d.	0.8 ± 0.083	n.d.	0.064 ± 0.056	0.058 ± 0.018	0.032 ± 8.3 x 10^{-3}
AncTPSd-1 AltAll	n.d.	n.d.	n.d.	0.016 ± 8.8 x 10^{-3}	n.d.	0.8 ± 0.033	n.d.	0.015 ± 4.4 x 10^{-3}	0.094 ± 0.01	0.07 ± 0.025
AncTPSd-1.2	n.d.	n.d.	n.d.	0.1 ± 3.9 x 10^{-3}	n.d.	0.82 ± 0.02	n.d.	0.012 ± 2.2 x 10^{-3}	0.027 ± 6.9 x 10^{-3}	0.036 ± 0.011
M1-1	1 ± 0	n.d.	n.d.	n.d.	n.d.	n.d.	n.d.	n.d.	n.d.	n.d.
M1-2	0.054 ± 9.2 x 10^{-3}	n.d.	n.d.	0.86 ± 0.023	n.d.	0.037 ± 0.012	n.d.	n.d.	0.052 ± 0.015	n.d.
AncTPSd-2	n.d.	n.d.	n.d.	0.053 ± 3.2 x 10^{-3}	n.d.	0.89 ± 0.045	n.d.	0.013 ± 0.011	0.023 ± 0.02	0.017 ± 0.015
AncTPSd-2 AltAll	n.d.	n.d.	n.d.	0.026 ± 7.7 x 10^{-3}	n.d.	0.85 ± 0.011	n.d.	0.013 ± 3.1 x 10^{-3}	0.066 ± 1.4 x 10^{-3}	0.043 ± 7.2 x 10^{-3}
AncTPSd-2.2	n.d.	n.d.	n.d.	0.082 ± 0.019	n.d.	0.84 ± 0.033	n.d.	7.6 x 10^{-3} ± 6.6 x 10^{-3}	0.03 ± 0.019	0.042 ± 9.7 x 10^{-3}
M2-1	0.11 ± 9.9 x 10^{-3}	n.d.	0.016 ± 5.8 x 10^{-4}	0.82 ± 0.01	0.054 ± 6.4 x 10^{-3}	n.d.	n.d.	n.d.	n.d.	n.d.
M2-2	0.32 ± 0.18	n.d.	0.035 ± 0.014	0.52 ± 0.24	0.07 ± 0.078	0.059 ± 0.023	n.d.	n.d.	n.d.	n.d.
AncTPSd-3	n.d.	n.d.	n.d.	0.063 ± 2.6 x 10^{-3}	n.d.	0.44 ± 0.081	n.d.	0.046 ± 0.023	0.31 ± 0.087	0.14 ± 0.028
AncTPSd-3 AltAlpha	n.d.	n.d.	n.d.	0.15 ± 9.9 x 10^{-3}	n.d.	0.28 ± 9.1 x 10^{-3}	n.d.	1.1 x 10^{-3} ± 2.5 x 10^{-3}	0.34 ± 0.013	0.22 ± 9.8 x 10^{-3}
AncTPSd-3.2	n.d.	n.d.	n.d.	0.25 ± 6.6 x 10^{-3}	6.9 x 10^{-3} ± 1.8 x 10^{-3}	0.29 ± 7.4 x 10^{-3}	n.d.	0.014 ± 1.1 x 10^{-3}	0.27 ± 9.4 x 10^{-3}	0.17 ± 4.8 x 10^{-3}
M3-1	0.39 ± 0.038	n.d.	n.d.	0.47 ± 0.034	0.14 ± 5.5 x 10^{-3}	n.d.	n.d.	n.d.	n.d.	n.d.
M3-2	0.076 ± 3.2 x 10^{-3}	n.d.	n.d.	0.87 ± 0.01	0.051 ± 7.1 x 10^{-3}	n.d.	n.d.	n.d.	n.d.	n.d.
AncTPSd-4	n.d.	5.7 x 10^{-4} ± 9.8 x 10^{-4}	n.d.	0.068 ± 9.4 x 10^{-3}	n.d.	0.32 ± 0.029	n.d.	0.021 ± 8.1 x 10^{-3}	0.38 ± 0.066	0.2 ± 0.034
AncTPSd-4 AltAlpha	n.d.	n.d.	n.d.	0.073 ± 9.0 x 10^{-3}	n.d.	0.35 ± 7.4 x 10^{-3}	n.d.	0.013 ± 1.2 x 10^{-3}	0.34 ± 0.014	0.23 ± 3.2 x 10^{-3}
M4-1	0.43 ± 0.014	n.d.	0.048 ± 6.5 x 10^{-3}	0.36 ± 0.021	0.15 ± 2.0 x 10^{-3}	n.d.	n.d.	n.d.	n.d.	n.d.
M4-2	0.29 ± 0.028	n.d.	n.d.	0.63 ± 0.028	0.078 ± 8.0 x 10^{-3}	n.d.	n.d.	n.d.	n.d.	n.d.
AncTPSd-5	n.d.	8.7 x 10^{-4} ± 2.0 x 10^{-3}	n.d.	0.065 ± 4.7 x 10^{-3}	n.d.	0.27 ± 0.054	n.d.	0.029 ± 0.019	0.46 ± 0.088	0.17 ± 0.048
AncTPSd-5 AltAlpha	n.d.	n.d.	n.d.	0.06 ± 4.1 x 10^{-3}	n.d.	0.32 ± 5.1 x 10^{-3}	n.d.	3.6 x 10^{-3} ± 7.8 x 10^{-4}	0.37 ± 1.7 x 10^{-3}	0.25 ± 3.4 x 10^{-3}
M5-1	0.32 ± 0.024	n.d.	n.d.	0.39 ± 6.7 x 10^{-3}	0.29 ± 0.025	n.d.	n.d.	n.d.	n.d.	n.d.
M5-2	n.d.	n.d.	n.d.	0.86 ± 6.7 x 10^{-3}	0.14 ± 6.7 x 10^{-3}	n.d.	n.d.	n.d.	n.d.	n.d.
M5-3	n.d.	0.32 ± 8.3 x 10^{-3}	n.d.	0.65 ± 8.6 x 10^{-3}	0.027 ± 7.9 x 10^{-4}	n.d.	n.d.	n.d.	n.d.	n.d.
M5-4	n.d.	0.051 ± 5.4 x 10^{-3}	n.d.	0.6 ± 9.3 x 10^{-3}	0.35 ± 4.0 x 10^{-3}	n.d.	n.d.	n.d.	n.d.	n.d.
M5-5	n.d.	n.d.	n.d.	0.16 ± 9.1 x 10^{-3}	n.d.	0.29 ± 7.8 x 10^{-3}	0.073 ± 2.1 x 10^{-3}	n.d.	0.3 ± 0.013	0.18 ± 0.012
M5-6	n.d.	0.023 ± 0.025	n.d.	0.37 ± 0.054	0.065 ± 6.0 x 10^{-3}	0.2 ± 0.033	n.d.	9.4 x 10^{-3} ± 0.01	0.21 ± 0.02	0.13 ± 0.021
M5-7	n.d.	n.d.	n.d.	0.35 ± 0.038	n.d.	0.22 ± 0.016	0.023 ± 0.013	n.d.	0.24 ± 0.019	0.17 ± 0.016
M5-8	n.d.	n.d.	n.d.	0.88 ± 0.056	n.d.	0.036 ± 0.011	6.6 x 10^{-3} ± 7.2 x 10^{-3}	n.d.	0.033 ± 9.3 x 10^{-3}	0.048 ± 0.029
M5-9	n.d.	5.8 x 10^{-3} ± 6.4 x 10^{-3}	n.d.	0.82 ± 0.061	0.011 ± 6.0 x 10^{-3}	0.046 ± 0.014	5.7 x 10^{-3} ± 6.3 x 10^{-3}	n.d.	0.05 ± 0.013	0.061 ± 0.031
M5-10	n.d.	n.d.	n.d.	0.5 ± 0.015	n.d.	0.098 ± 0.023	0.14 ± 0.015	n.d.	0.14 ± 0.046	0.12 ± 9.0 x 10^{-3}
M5-11	n.d.	n.d.	n.d.	0.94 ± 0.066	n.d.	0.01 ± 0.011	7.4 x 10^{-3} ± 8.1 x 10^{-3}	n.d.	0.011 ± 0.012	0.032 ± 0.035
M5-12	n.d.	0.18 ± 4.9 x 10^{-3}	n.d.	0.79 ± 5.3 x 10^{-3}	0.031 ± 9.1 x 10^{-3}	n.d.	n.d.	n.d.	n.d.	n.d.
M5-13	n.d.	0.41 ± 5.2 x 10^{-3}	n.d.	0.54 ± 6.4 x 10^{-3}	0.049 ± 4.3 x 10^{-3}	n.d.	n.d.	n.d.	n.d.	n.d.
M5-14	n.d.	0.73 ± 0.012	n.d.	0.16 ± 3.8 x 10^{-3}	0.11 ± 8.6 x 10^{-3}	n.d.	n.d.	n.d.	n.d.	n.d.
M5-15	n.d.	0.2 ± 4.0 x 10^{-3}	0.018 ± 3.7 x 10^{-3}	0.7 ± 3.8 x 10^{-3}	0.076 ± 3.5 x 10^{-3}	n.d.	n.d.	n.d.	n.d.	n.d.
M5-16	n.d.	0.77 ± 6.4 x 10^{-3}	n.d.	0.067 ± 1.2 x 10^{-3}	0.16 ± 5.2 x 10^{-3}	n.d.	n.d.	n.d.	n.d.	n.d.
M5-17	n.d.	0.45 ± 1.6 x 10^{-3}	n.d.	0.37 ± 4.7 x 10^{-4}	0.18 ± 1.3 x 10^{-3}	n.d.	n.d.	n.d.	n.d.	n.d.
M5-18	n.d.	0.35 ± 3.6 x 10^{-3}	n.d.	0.28 ± 9.0 x 10^{-4}	0.37 ± 2.8 x 10^{-3}	n.d.	n.d.	n.d.	n.d.	n.d.
M5-19	n.d.	0.026 ± 5.4 x 10^{-3}	n.d.	0.79 ± 4.7 x 10^{-3}	0.19 ± 1.2 x 10^{-3}	n.d.	n.d.	n.d.	n.d.	n.d.
M5-20	n.d.	0.019 ± 7.0 x 10^{-3}	n.d.	0.52 ± 1.2 x 10^{-3}	0.46 ± 8.0 x 10^{-3}	n.d.	n.d.	n.d.	n.d.	n.d.
M5-21	n.d.	0.16 ± 7.9 x 10^{-3}	n.d.	0.32 ± 0.012	0.52 ± 0.013	n.d.	n.d.	n.d.	n.d.	n.d.
M5-22	n.d.	0.011 ± 5.4 x 10^{-4}	n.d.	0.36 ± 2.4 x 10^{-3}	0.63 ± 2.6 x 10^{-3}	n.d.	n.d.	n.d.	n.d.	n.d.
M5-23	n.d.	0.075 ± 5.0 x 10^{-3}	n.d.	0.27 ± 7.4 x 10^{-3}	0.65 ± 9.5 x 10^{-3}	n.d.	n.d.	n.d.	n.d.	n.d.
M5-24	n.d.	0.02 ± 4.2 x 10^{-3}	n.d.	0.46 ± 1.8 x 10^{-3}	0.52 ± 3.8 x 10^{-3}	n.d.	n.d.	n.d.	n.d.	n.d.
M5-25	n.d.	n.d.	n.d.	0.082 ± 2.8 x 10^{-3}	0.92 ± 2.8 x 10^{-3}	n.d.	n.d.	n.d.	n.d.	n.d.
AncTPSd-6	n.d.	n.d.	n.d.	0.021 ± 4.8 x 10^{-3}	1.1 x 10^{-3} ± 1.3 x 10^{-3}	0.29 ± 0.028	n.d.	9.8 x 10^{-3} ± 8.0 x 10^{-3}	0.45 ± 0.044	0.22 ± 0.038
M6-1	n.d.	n.d.	n.d.	0.095 ± 2.6 x 10^{-3}	0.9 ± 2.6 x 10^{-3}	n.d.	n.d.	n.d.	n.d.	n.d.
M6-2	n.d.	n.d.	n.d.	0.35 ± 3.9 x 10^{-3}	0.61 ± 6.4 x 10^{-3}	0.015 ± 1.0 x 10^{-3}	n.d.	n.d.	0.014 ± 1.9 x 10^{-3}	0.018 ± 2.0 x 10^{-3}
M6-3	n.d.	0.089 ± 0.051	n.d.	0.7 ± 0.019	0.019 ± 8.1 x 10^{-4}	0.053 ± 0.011	n.d.	3.6 x 10^{-3} ± 2.6 x 10^{-3}	0.084 ± 0.012	0.053 ± 0.011
M6-5	n.d.	n.d.	n.d.	0.1 ± 9.7 x 10^{-3}	0.074 ± 0.012	0.24 ± 0.013	n.d.	0.016 ± 0.013	0.34 ± 0.018	0.23 ± 4.2 x 10^{-3}
AncTPSd-7	n.d.	n.d.	n.d.	0.015 ± 3.5 x 10^{-3}	n.d.	0.3 ± 0.048	n.d.	9.7 x 10^{-3} ± 9.0 x 10^{-3}	0.43 ± 0.028	0.25 ± 9.3 x 10^{-3}
M7-1	n.d.	n.d.	n.d.	0.072 ± 4.3 x 10^{-3}	0.93 ± 4.3 x 10^{-3}	n.d.	n.d.	n.d.	n.d.	n.d.
M7-2	n.d.	n.d.	n.d.	0.27 ± 8.0 x 10^{-3}	0.68 ± 6.9 x 10^{-3}	0.011 ± 3.9 x 10^{-3}	n.d.	n.d.	0.012 ± 2.6 x 10^{-3}	0.018 ± 2.0 x 10^{-3}
M7-3	n.d.	0.097 ± 0.068	n.d.	0.77 ± 0.056	0.022 ± 2.7 x 10^{-3}	0.021 ± 5.9 x 10^{-3}	n.d.	1.2 x 10^{-3} ± 1.3 x 10^{-3}	0.052 ± 5.4 x 10^{-3}	0.032 ± 4.1 x 10^{-3}
M7-5	n.d.	n.d.	n.d.	0.15 ± 0.014	0.082 ± 5.5 x 10^{-3}	0.18 ± 4.1 x 10^{-3}	n.d.	0.021 ± 4.2 x 10^{-3}	0.31 ± 8.9 x 10^{-3}	0.26 ± 0.011
AncTPSd-8	n.d.	n.d.	n.d.	0.022 ± 5.2 x 10^{-3}	n.d.	0.27 ± 0.052	n.d.	0.02 ± 0.019	0.48 ± 0.071	0.21 ± 0.055
AncTPSd-8 AltAlpha	n.d.	n.d.	n.d.	0.014 ± 4.7 x 10^{-3}	n.d.	0.31 ± 0.022	n.d.	n.d.	0.41 ± 0.014	0.27 ± 0.024
M8-1	n.d.	n.d.	n.d.	0.15 ± 0.067	0.85 ± 0.067	n.d.	n.d.	n.d.	n.d.	n.d.
M8-2	n.d.	n.d.	n.d.	0.36 ± 6.5 x 10^{-3}	0.64 ± 6.5 x 10^{-3}	n.d.	n.d.	n.d.	n.d.	n.d.
M8-3	n.d.	0.12 ± 0.057	n.d.	0.7 ± 0.038	0.022 ± 3.7 x 10^{-3}	0.034 ± 9.3 x 10^{-3}	n.d.	2.1 x 10^{-3} ± 2.7 x 10^{-3}	0.068 ± 9.2 x 10^{-3}	0.045 ± 6.7 x 10^{-3}
M8-5	n.d.	n.d.	n.d.	0.21 ± 0.025	0.09 ± 7.0 x 10^{-3}	0.21 ± 0.018	n.d.	0.03 ± 1.8 x 10^{-3}	0.22 ± 0.014	0.25 ± 0.018
AncTPSd-9	n.d.	n.d.	n.d.	0.94 ± 0.012	n.d.	0.014 ± 8.2 x 10^{-3}	n.d.	9.5 x 10^{-3} ± 9.2 x 10^{-4}	0.017 ± 4.7 x 10^{-3}	0.015 ± 8.2 x 10^{-3}
AncTPSd-9 AltAlpha	n.d.	n.d.	n.d.	0.95 ± 0.016	4.3 x 10^{-3} ± 2.9 x 10^{-3}	7.5 x 10^{-3} ± 2.2 x 10^{-3}	n.d.	2.3 x 10^{-3} ± 3.9 x 10^{-3}	0.015 ± 1.4 x 10^{-3}	0.016 ± 7.1 x 10^{-3}
M9-1	n.d.	n.d.	n.d.	0.48 ± 3.9 x 10^{-3}	0.52 ± 3.9 x 10^{-3}	n.d.	n.d.	n.d.	n.d.	n.d.
M9-2	n.d.	n.d.	n.d.	0.97 ± 2.1 x 10^{-3}	0.035 ± 2.1 x 10^{-3}	n.d.	n.d.	n.d.	n.d.	n.d.
M9-3	n.d.	0.22 ± 0.13	n.d.	0.33 ± 0.05	0.46 ± 0.079	n.d.	n.d.	n.d.	n.d.	n.d.
M9-5	n.d.	n.d.	n.d.	0.088 ± 8.6 x 10^{-3}	0.91 ± 8.6 x 10^{-3}	n.d.	n.d.	n.d.	n.d.	n.d.
M9-6	n.d.	n.d.	n.d.	1 ± 0	n.d.	n.d.	n.d.	n.d.	n.d.	n.d.
M9-8	n.d.	0.17 ± 0.043	n.d.	0.58 ± 0.038	0.25 ± 0.015	n.d.	n.d.	n.d.	n.d.	n.d.
M9-9	n.d.	n.d.	n.d.	0.2 ± 8.4 x 10^{-3}	0.8 ± 8.4 x 10^{-3}	n.d.	n.d.	n.d.	n.d.	n.d.
AncTPSd-10	n.d.	n.d.	n.d.	0.14 ± 0.038	0.86 ± 0.038	n.d.	n.d.	n.d.	n.d.	n.d.
PaTPS-ISO	n.d.	n.d.	n.d.	n.d.	1 ± 0	n.d.	n.d.	n.d.	n.d.	n.d.
PaTPS-LAS	n.d.	n.d.	n.d.	0.022 ± 1.9 x 10^{-3}	n.d.	0.28 ± 0.071	n.d.	0.031 ± 0.032	0.48 ± 0.089	0.19 ± 0.049

Table S2. Ancestral TPS-d3 enzymes targeted for mutagenesis in this study, corresponding mutant letter and number codes, and sites targeted. *, sites replaced in the protein background. **, proteins that naturally contain the mutant residue(s). Numbering is according to the *Abies grandis* TPS-LAS (27). “Ab”, *A. balsamea*; “Pa”, *Picea abies*.

Wild-type target	Mutatnt ID	Type	Target residue(s)*	Mutation(s)	Source(s) of derived state**
AncTPS$_d$-1	M1-1	Single	723	Ala to Thr	AbTPS-ISO
AncTPS$_d$-1	M1-2	Single	723	Ala to Ser	PaTPS-ISO
AncTPS$_d$-2	M2-1	Single	723	Ala to Thr	AbTPS-ISO
AncTPS$_d$-2	M2-2	Single	723	Ala to Ser	PaTPS-ISO
AncTPS$_d$-3	M3-1	Single	723	Ala to Thr	AbTPS-ISO
AncTPS$_d$-3	M3-2	Single	723	Ala to Ser	PaTPS-ISO
AncTPS$_d$-4	M4-1	Single	723	Ala to Thr	AbTPS-ISO
AncTPS$_d$-4	M4-2	Single	723	Ala to Ser	PaTPS-ISO
AncTPS$_d$-5	M5-1	Single	723	Ala to Thr	AbTPS-ISO
AncTPS$_d$-5	M5-2	Single	723	Ala to Ser	PaTPS-ISO
AncTPS$_d$-5	M5-3	Single	724	Leu to Gly	AncTPS$_d$-10
AncTPS$_d$-5	M5-4	Double	724; 833	Leu to Gly; Thr to Tyr	AncTPS$_d$-10
AncTPS$_d$-5	M5-5	Chimera	591-694	Region X	AncTPS$_d$-9
AncTPS$_d$-5	M5-6	Chimera	695-755	Region Y	AncTPS$_d$-9
AncTPS$_d$-5	M5-7	Chimera	756-843	Region Z	AncTPS$_d$-9
AncTPS$_d$-5	M5-8	Chimera	591-755	Regions X, Y	AncTPS$_d$-9
AncTPS$_d$-5	M5-9	Chimera	695-843	Regions Y, Z	AncTPS$_d$-9
AncTPS$_d$-5	M5-10	Chimera	591-694; 756-843	Regions X, Z	AncTPS$_d$-9
AncTPS$_d$-5	M5-11	Chimera	591-843	Regions X, Y, Z	AncTPS$_d$-9
AncTPS$_d$-5	M5-12	Chimera, single	591-694; 724	Region X; Leu to Gly	AncTPS$_d$-9 (X), AncTPS$_d$-10 (Gly)
AncTPS$_d$-5	M5-13	Chimera, single	695-755; 724	Region Y; Leu to Gly	AncTPS$_d$-9 (Y), AncTPS$_d$-10 (Gly)
AncTPS$_d$-5	M5-14	Chimera, single	756-843; 724	Region Z; Leu to Gly	AncTPS$_d$-9 (Z), AncTPS$_d$-10 (Gly)
AncTPS$_d$-5	M5-15	Chimera, single	591-755; 724	Regions X, Y; Leu to Gly	AncTPS$_d$-9 (XY), AncTPS$_d$-10 (Gly)
AncTPS$_d$-5	M5-16	Chimera, single	695-843; 724	Regions Y, Z; Leu to Gly	AncTPS$_d$-9 (YZ), AncTPS$_d$-10 (Gly)
AncTPS$_d$-5	M5-17	Chimera, single	591-694; 756-843; 724	Regions X, Z; Leu to Gly	AncTPS$_d$-9 (XZ), AncTPS$_d$-10 (Gly)
AncTPS$_d$-5	M5-18	Chimera, single	591-843; 724	Regions X, Y, Z; Leu to Gly	AncTPS$_d$-9 (XYZ), AncTPS$_d$-10 (Gly)
AncTPS$_d$-5	M5-19	Chimera, double	591-694; 724; 833	Region X; Leu to Gly; Thr to Tyr	AncTPS$_d$-9 (X), AncTPS$_d$-10 (Gly, Tyr)
AncTPS$_d$-5	M5-20	Chimera, double	695-755; 724; 833	Region Y; Leu to Gly; Thr to Tyr	AncTPS$_d$-9 (Y), AncTPS$_d$-10 (Gly, Tyr)
AncTPS$_d$-5	M5-21	Chimera, double	756-843; 724; 833	Region Z; Leu to Gly; Asn to Tyr	AncTPS$_d$-9 (Z), AncTPS$_d$-10 (Gly, Tyr)
AncTPS$_d$-5	M5-22	Chimera, double	591-755; 724; 833	Regions X, Y; Leu to Gly; Thr to Tyr	AncTPS$_d$-9 (XY), AncTPS$_d$-10 (Gly, Tyr)
AncTPS$_d$-5	M5-23	Chimera, double	695-843; 724; 833	Regions Y, Z; Leu to Gly; Asn to Tyr	AncTPS$_d$-9 (YZ), AncTPS$_d$-10 (Gly, Tyr)
AncTPS$_d$-5	M5-24	Chimera, double	591-694; 756-843; 724; 833	Regions X, Z; Leu to Gly; Asn to Tyr	AncTPS$_d$-9 (XZ), AncTPS$_d$-10 (Gly, Tyr)
AncTPS$_d$-5	M5-25	Chimera, double	591-843; 724; 833	Regions X, Y, Z; Leu to Gly; Asn to Tyr	AncTPS$_d$-9 (XYZ), AncTPS$_d$-10 (Gly, Tyr)
AncTPS$_d$-6	M6-1	Single	723	Ala to Thr	AbTPS-ISO
AncTPS$_d$-6	M6-2	Single	723	Ala to Ser	PaTPS-ISO
AncTPS$_d$-6	M6-3	Single	724	Leu to Gly	AncTPS$_d$-10
AncTPS$_d$-6	M6-4	Single	833	Thr to Tyr	AncTPS$_d$-10
AncTPS$_d$-6	M6-5	Double	724; 833	Leu to Gly; Thr to Tyr	AncTPS$_d$-10
AncTPS$_d$-7	M7-1	Single	723	Ala to Thr	AbTPS-ISO
AncTPS$_d$-7	M7-2	Single	723	Ala to Ser	PaTPS-ISO
AncTPS$_d$-7	M7-3	Single	724	Leu to Gly	AncTPS$_d$-10
AncTPS$_d$-7	M7-4	Single	833	Thr to Tyr	AncTPS$_d$-10
AncTPS$_d$-7	M7-5	Double	724; 833	Leu to Gly; Thr to Tyr	AncTPS$_d$-10
AncTPS$_d$-8	M8-1	Single	723	Ala to Thr	AbTPS-ISO
AncTPS$_d$-8	M8-2	Single	723	Ala to Ser	PaTPS-ISO
AncTPS$_d$-8	M8-3	Single	724	Leu to Gly	AncTPS$_d$-10
AncTPS$_d$-8	M8-4	Single	833	Thr to Tyr	AncTPS$_d$-10
AncTPS$_d$-8	M8-5	Double	724; 833	Leu to Gly; Thr to Tyr	AncTPS$_d$-10
AncTPS$_d$-9	M9-1	Single	723	Ala to Thr	AbTPS-ISO
AncTPS$_d$-9	M9-2	Single	723	Ala to Ser	PaTPS-ISO
AncTPS$_d$-9	M9-3	Single	724	Leu to Gly	AncTPS$_d$-10
AncTPS$_d$-9	M9-4	Single	833	Asn to Tyr	AncTPS$_d$-10
AncTPS$_d$-9	M9-5	Double	724; 833	Leu to Gly; Asn to Tyr	AncTPS$_d$-10
AncTPS$_d$-9	M9-6	Chimera	591-694	Region X	AncTPS$_d$-10
AncTPS$_d$-9	M9-7	Chimera	756-843	Region Z	AncTPS$_d$-10
AncTPS$_d$-9	M9-8	Chimera	591-755	Regions X, Y	AncTPS$_d$-10
AncTPS$_d$-9	M9-9	Chimera	591-843	Regions X, Y, Z	AncTPS$_d$-10

Table S3. Distance matrix of ancestral and selected extant TPS-d3 alpha domains. Each pairwise comparison shows the total number of amino acid residue differences (“Hamming” method with a gap penalty of 1). The alpha domain spans from residues 559 through 868 as numbered according to *Abies grandis* TPS-LAS (27). Percent identities (alpha domain only) are shown in parentheses. “Ab”; *A. balsamea*; “Pa”, *Picea abies*.

**Enzyme**	**AgTPS-LAS**	**AbTPS-ISO**	**PaTPS-LAS**	**PaTPS-ISO**	**AncTPS$_d$-10**	**AncTPS$_d$-9**	**AncTPS$_d$-8**	**AncTPS$_d$-7**	**AncTPS$_d$-6**	**AncTPS$_d$-5**	**AncTPS$_d$-4**	**AncTPS$_d$-3**	**AncTPS$_d$-2**
AbTPS-ISO	55 (82.6)												
PaTPS-LAS	46 (85.5)	61 (80.4)											
PaTPS-ISO	59 (81.3)	64 (79.4)	30 (90.4)										
AncTPS$_d$-10	93 (70.3)	96 (69.1)	91 (70.7)	92 (70.4)									
AncTPS$_d$-9	83 (73.5)	84 (73)	78 (74.9)	78 (74.9)	20 (93.6)								
AncTPS$_d$-8	23 (92.9)	36 (88.4)	28 (91)	45 (85.5)	80 (74.3)	67 (78.5)							
AncTPS$_d$-7	51 (83.9)	69 (77.8)	34 (89.1)	47 (84.9)	97 (68.8)	84 (73)	40 (87.1)						
AncTPS$_d$-6	41 (87.1)	57 (81.7)	6 (98.1)	24 (92.3)	85 (72.7)	72 (76.8)	24 (92.3)	29 (90.7)					
AncTPS$_d$-5	56 (82.3)	57 (81.7)	49 (84.2)	52 (83.3)	49 (84.2)	34 (89.1)	35 (88.7)	55 (82.3)	43 (86.2)				
AncTPS$_d$-4	62 (80.3)	61 (80.4)	54 (82.6)	57 (81.7)	58 (81.4)	43 (86.2)	41 (86.8)	62 (80.1)	48 (84.6)	10 (96.8)			
AncTPS$_d$-3	74 (76.7)	69 (78.1)	63 (80)	66 (79)	68 (78.4)	55 (82.6)	55 (82.6)	72 (77.1)	58 (81.6)	24 (92.6)	16 (95.2)		
AncTPS$_d$-2	106 (66.3)	101 (67.7)	101 (67.7)	104 (66.8)	104 (66.8)	96 (69.4)	91 (71)	99 (68.4)	97 (69)	73 (76.8)	66 (79)	54 (83.2)	
AncTPS$_d$-1	121 (61.5)	119 (61.9)	121 (61.3)	124 (60.3)	120 (61.6)	115 (63.2)	112 (64.2)	120 (61.6)	117 (62.6)	99 (68.4)	96 (69.4)	84 (73.5)	43 (86.1)

Table S4. Diterpene olefin abundances for ancestral and mutant TPSs characterized in *E. coli*. WT, "wild-type" ancestral TPS; *, total diterpene abundance was calculated as the total peak area (log_{10}) of class-I cyclization products. The abundance (log_{10}) of isopimaradiene is also shown. n.d., not detected.

Construct name	Total diterpenes*	isopimaradiene
$AncTPS_d$ -1 (WT)	6.250	n.d.
M1-1	4.855	n.d.
M1-2	5.816	n.d.
$AncTPS_d$ -2 (WT)	5.967	n.d.
M2-1	6.104	4.845
M2-2	5.876	4.653
$AncTPS_d$ -3 (WT)	7.455	n.d.
M3-1	6.165	5.312
M3-2	6.133	4.845
$AncTPS_d$ -4 (WT)	6.831	n.d.
M4-1	5.677	4.858
M4-2	5.945	4.822
$AncTPS_d$ -5 (WT)	6.915	n.d.
M5-1	5.155	4.621
M5-2	5.736	4.899

Table S5. Diterpene olefin abundances for ancestral and mutant TPSs characterized *in vitro*. WT, "wild-type" ancestral TPS; *, total diterpene abundance was calculated as the total peak area (log_{10}) of class-I cyclization products. The abundance (log_{10}) of isopimaradiene is also shown. n.d., not detected. Pa, *Picea abies*.

Construct name	Total diterpenes*	isopimaradiene
$AncTPS_d$ -1 (WT)	7.585	n.d.
$AncTPS_d$ -1 AltAll	7.362	n.d.
$AncTPS_d$ -1.2	7.308	n.d.
$AncTPS_d$ -2 (WT)	7.534	n.d.
$AncTPS_d$ -2 AltAll	7.585	n.d.
$AncTPS_d$ -2.2	7.129	n.d.
$AncTPS_d$ -3 (WT)	7.738	n.d.
$AncTPS_d$ -3 AltAlpha	7.608	n.d.
$AncTPS_d$ -3.2	7.509	5.354
$AncTPS_d$ -4 (WT)	7.808	n.d.
$AncTPS_d$ -4 AltAlpha	7.709	n.d.
$AncTPS_d$ -5 (WT)	7.796	n.d.
$AncTPS_d$ -5 AltAlpha	7.725	n.d.
M5-3	7.460	5.896
M5-4	7.806	7.348
M5-5	8.046	n.d.
M5-6	7.895	6.700
M5-7	7.916	n.d.
M5-8	7.976	n.d.
M5-9	7.937	6.055
M5-10	7.893	n.d.
M5-11	7.977	n.d.
M5-12	7.976	6.490
M5-13	7.507	6.201
M5-14	7.848	6.888
M5-15	7.992	6.869
M5-16	7.718	6.921
M5-17	8.109	7.353
M5-18	7.936	7.500
M5-19	7.934	7.208
M5-20	7.162	6.826
M5-21	7.267	6.985
M5-22	8.109	7.907
M5-23	7.765	7.581
M5-24	8.083	7.801
M5-25	8.088	8.051
$AncTPS_d$ -6 (WT)	7.872	4.826
M6-1	7.453	7.410
M6-2	8.081	7.863
M6-3	8.010	6.297
M6-5	7.647	6.509
$AncTPS_d$ -7 (WT)	7.886	n.d.
M7-1	7.862	7.830
M7-2	8.178	8.013
M7-3	8.125	6.472
M7-5	6.905	5.819
$AncTPS_d$ -8 (WT)	7.897	n.d.
$AncTPS_d$ -8 AltAlpha	7.108	n.d.
M8-1	6.849	6.781
M8-2	7.723	7.527
M8-3	7.752	6.088
M8-5	6.913	5.870
$AncTPS_d$ -9 (WT)	8.000	n.d.
$AncTPS_d$ -9 AltAlpha	7.556	5.206
M9-1	7.958	7.675
M9-2	8.010	6.555
M9-3	7.783	7.435
M9-5	7.720	7.681
M9-6	8.012	n.d.
M9-8	7.462	6.860
M9-9	7.531	7.436
$AncTPS_d$ -10	7.801	7.728
PaTPS-ISO	6.444	6.444
PaTPS-LAS	7.507	n.d.

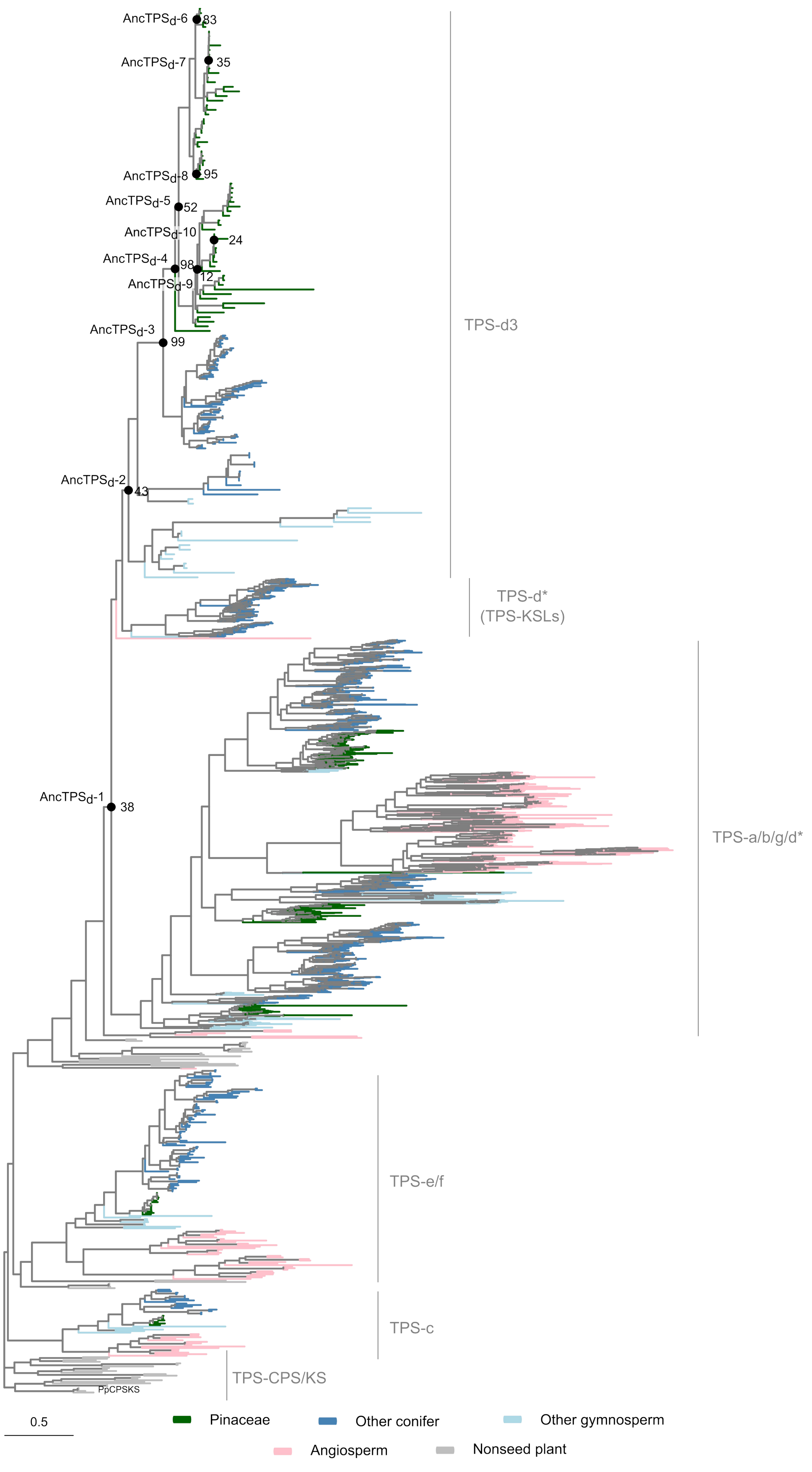

Fig. S1. Phylogenetic positions of ancestral TPS-d3 enzymes among land plant terpene synthases. A local BLAST library was constructed consisting of sequences from representative land plant genomes and transcriptomes and seeded with selected characterized terpene synthases from land plants. Maximum-likelihood phylogenetic analysis was performed using ca. 1,600 sequences and the resulting best tree is shown. Tip branches are colored according to the plant clade shown in the legend (bottom). Bootstrap support values at nodes equivalent to the 10 ancestral sequences analyzed in this study are shown. *, clade contains sequences designated as either “TPS-d3” or simply “TPS-d” in at least one other study, but are considered to be a different subfamily here for clarity.

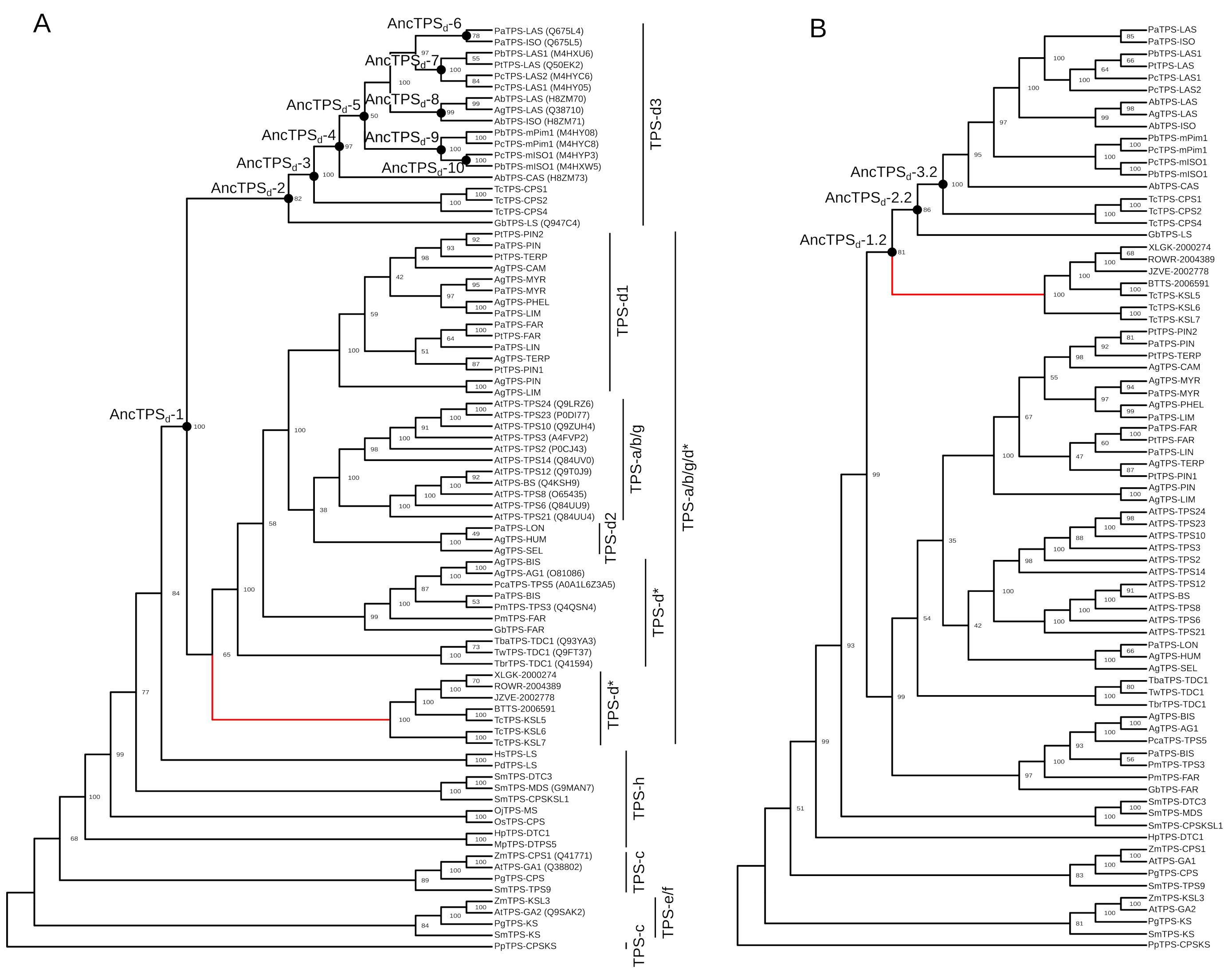

Fig. S2. Phylogenetic trees used in estimation of ancestral sequences and TPS subfamily designations as used in this study. (*A*) Phylogenetic tree used to estimate AncTPS$_d$-1 through AncTPS$_d$-10, as well as alternative ancestral estimates. *; clade contains sequences referred to previously as either "TPS-d3" or simply "TPS-d" enzymes, but are not considered as TPS-d3 enzymes here for clarity . (*B*) Alternative phylogenetic tree used to estimate the sequences of AncTPS$_d$-1.2, AncTPS$_d$-2.2, and AncTPS$_d$-3.2. In (*A*) and (*B*), the red branch had variable placement and might affect ancestral estimates near the TPS-d3 origin. Bootstrap support values for branches are shown at internal nodes. Sequences retrieved from Uniprot have sequence IDs in parentheses, and remaining sequences were retrieved from Genbank and have the following accession numbers: TcTPS-CPS1, KT588482; TcTPS-CPS2, KT588483; TcTPS-CPS4, JN587309; PtTPS-PIN2, AF543527; PaTPS-PIN, AY473622; PtTPS-TERP, AF543529; AgTPS-CAM, U87910; AgTPS-MYR, U87908; PaTPS-MYR, AY473626; AgTPS-PHEL, AF139205; PaTPS-LIM, AY473624; PaTPS-FAR, AY473627; PtTPS-FAR, AF543528; PaTPS-LIN, AY473623; AgTPS-TERP, AF139206; PtTPS-PIN1, AF543530; AgTPS-PIN, AF139207; AgTPS-LIM, AF006193; PaTPS-LON, AY473625; AgTPS-HUM, U92267; AgTPS-SEL, U92266; AgTPS-BIS, AF006195; PaTPS-BIS, AY473619; PmTPS-FAR, HQ214483; GbTPS-FAR, KM248383; TcTPS-KSL5, KT588490; TcTPS-KSL6, KT588487; TcTPS-KSL7, KT588488; HsTPS-LS, OL989441; PdTPS-LS, OL989442; SmTPS-DTC3, AB898932; SmTPS-CPSKSL1, JN001323; OjTPS-MS, OL989443; OsTPS-CPS, OL989450; HpTPS-DTC1, LC128408; MpTPS-DTPS5, OL989446; PgTPS-CPS, GU045755; SmTPS-TPS9, JX413782; ZmTPS-KSL3, DAA36069; PgTPS-KS, GU045756; SmTPS-KS, AB898933; PpTPS-CPSKS, AB302933.

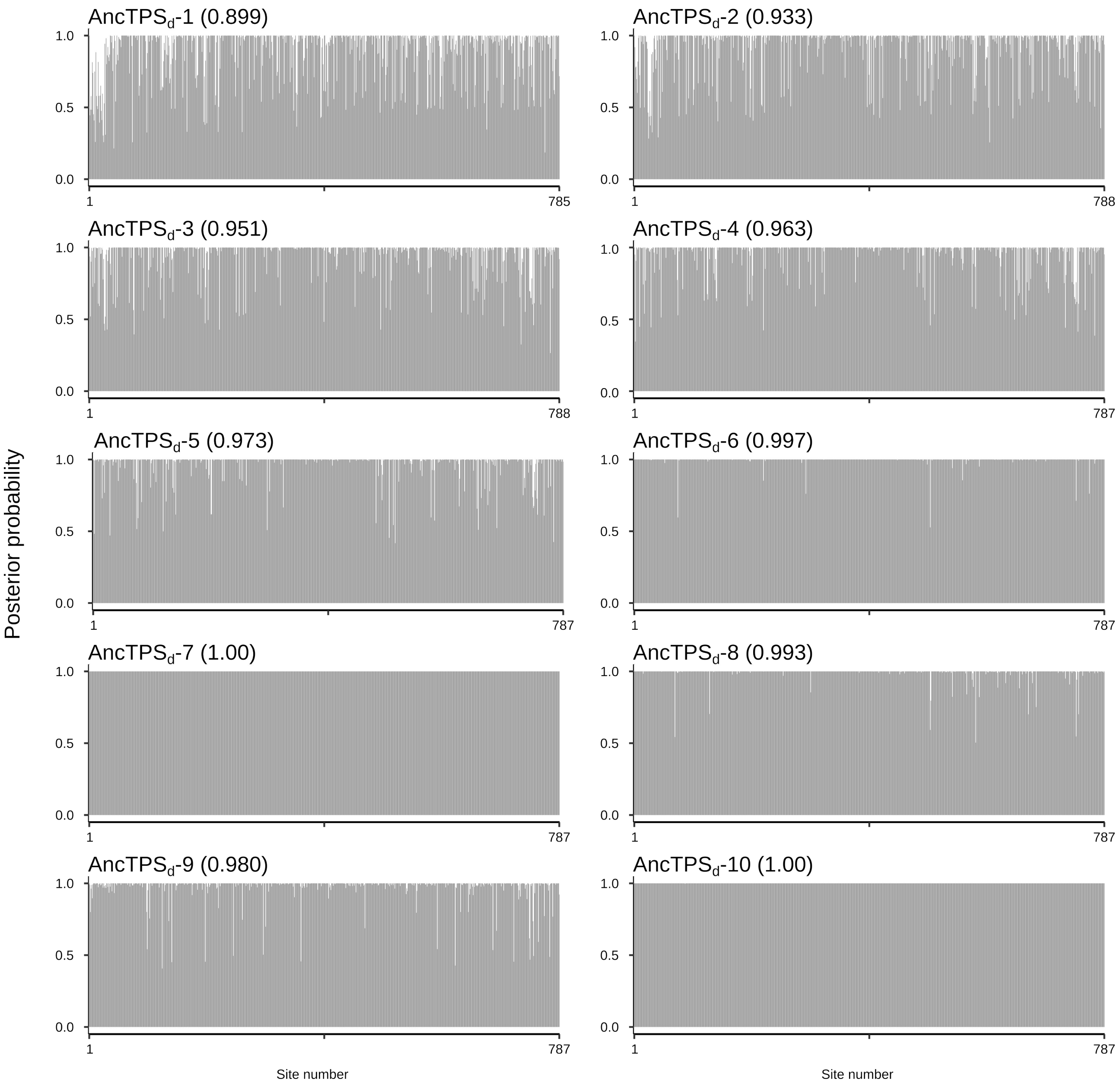

Fig. S3. Site-by-site posterior probabilities of ancestral sequences. Histograms show the probability of the most likely amino acid at each residue position of the 10 ancestral TPS-d3 enzymes selected for analysis in this study. The average posterior probability across all sites is shown in parentheses. Site numbers correspond directly to the ancestral protein sequences.

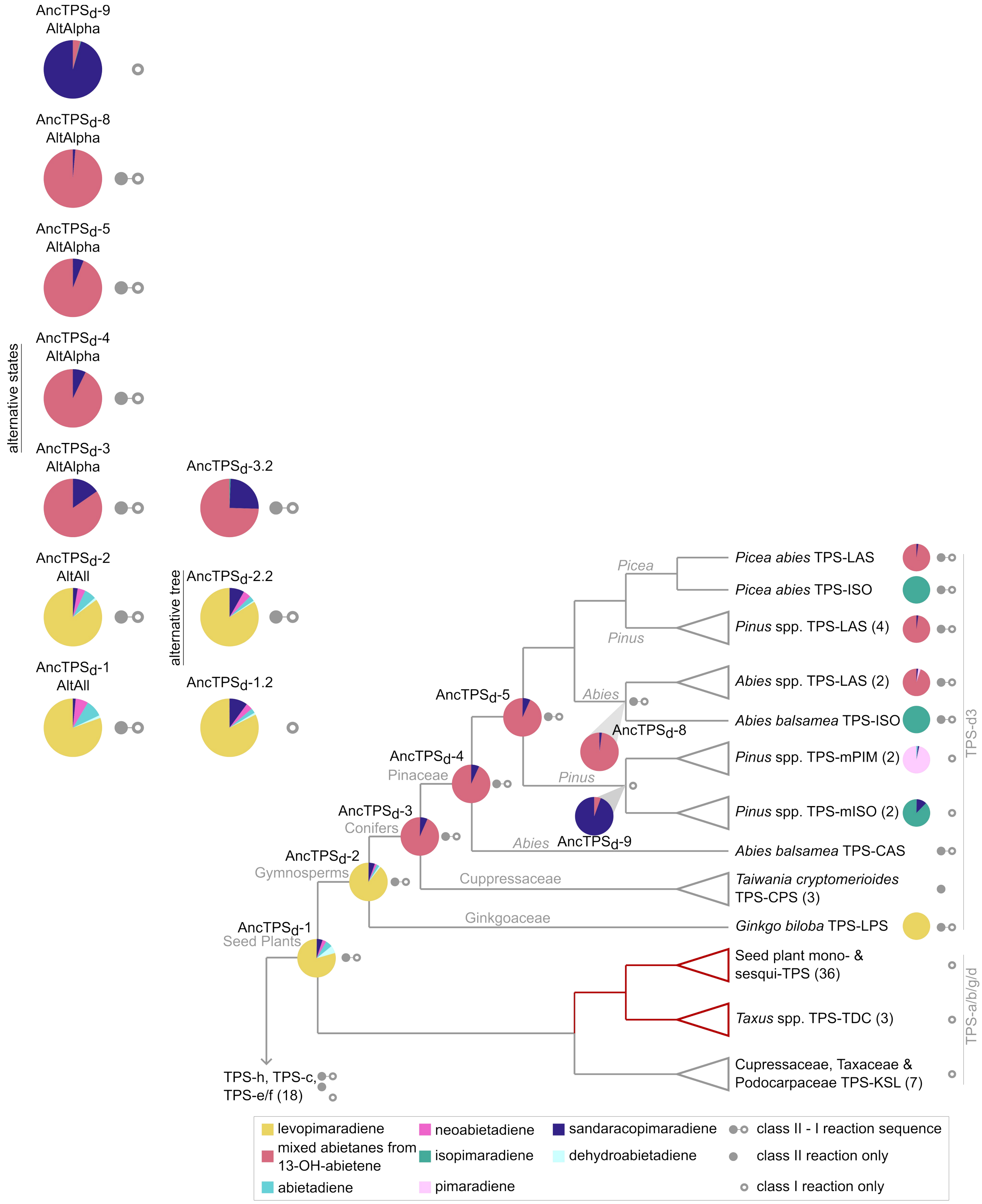

Fig. S4. Activities of alternative ancestral enzymes. The effects of uncertainty in the primary ancestral sequence estimates were evaluated using the relative product abundances of 7 alternative ancestral TPS-d3 sequences ("AltAll" and "AltAlpha" variants, pie charts in the upper-left). The effect of phylogenetic uncertainty caused by variable placement of "kaurene-synthase-like" (KSL) enzymes was evaluated by testing the activities of three ancestral sequences estimated from a different tree (shown in Fig. S2) ($AncTPS_d$-1.2 through $AncTPS_d$-3.2, upper-left). Red branches indicate terpene synthase subfamilies that are not inferred to be descendants of $AncTPS_d$-1.2 in the alternative phylogeny.

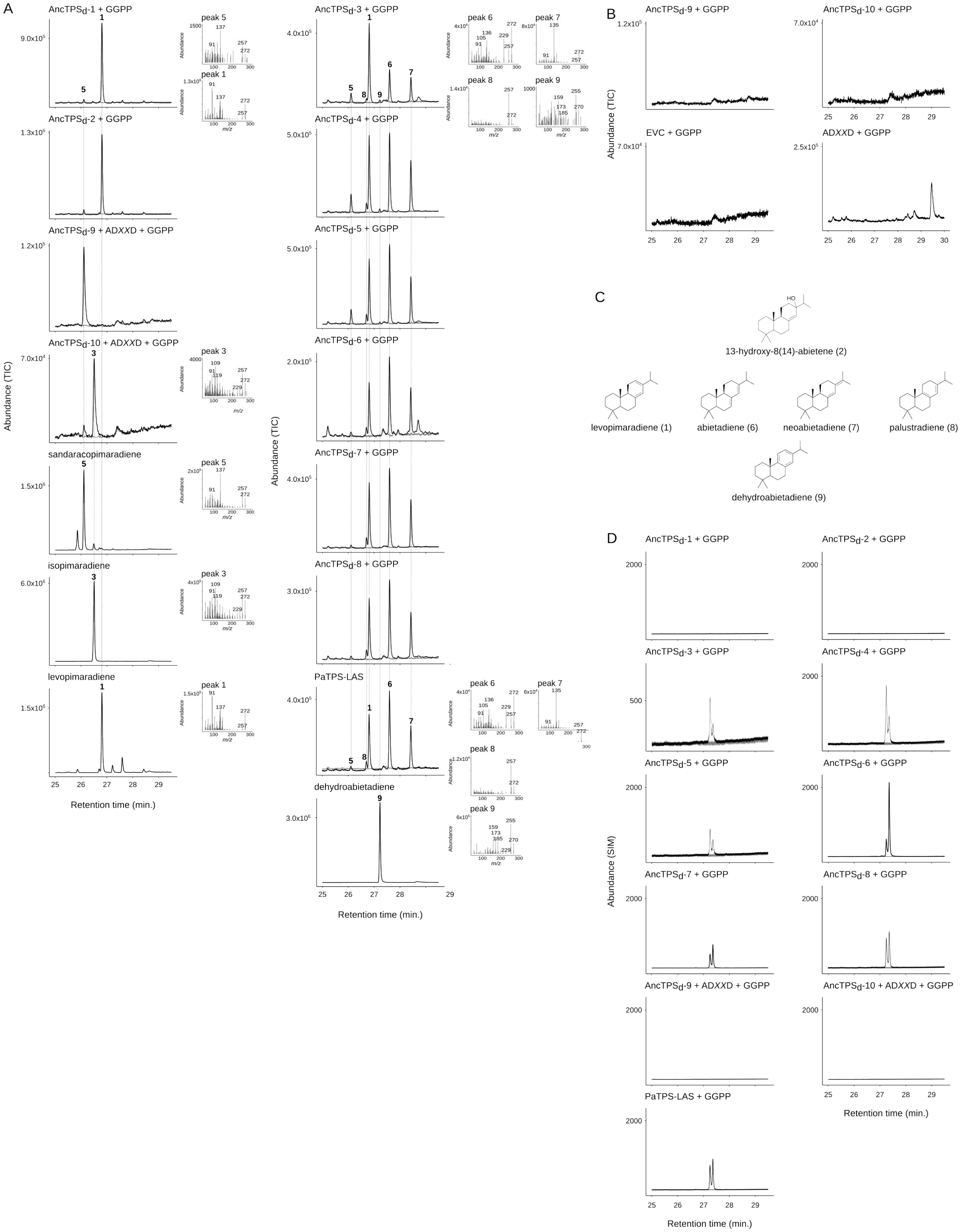

Fig. S5. Gas chromatography-mass spectrometry results of ancestral TPS-d3 enzymes. (*A*) representative total ion chromatograms (TIC) and mass spectra for the products identified in ancestral enzyme assays. In each assay, either geranylgeranyl diphosphate (GGPP) standard was supplied, or (+)-copalyl diphosphate (CPP) was supplied to ancestral enzymes through enzymatic conversion of GGPP using a class-I knockout of AncTPS$_d$-8 ("AD*XX*D"). All products were identified through comparisons of retention times and mass spectra to authentic standards. The multiple previously described abietane peaks produced by *Picea abies* TPS-LAS (PaTPS-LAS) (22) are shown instead of standards for brevity. Mass spectra for the first occurrence of each diterpene product are shown to the right of chromatograms. (*B*) Chromatograms showing that no diterpene olefin products are detected when GGPP is assayed with AncTPS$_d$-9, AncTPS$_d$-10, the background of an *Escherichia coli* empty vector control (EVC), nor with the AD*XX*D construct. The AD*XX*D construct contained an additional peak near 29.5 minutes, which we presume to be CPP after endogenous diphosphate removal in the *E. coli* background. (*C*) The major products of TPS-LAS enzymes, epimers of 13-hydroxy-8(14)-abietene, rearrange non-enzymatically to multiple abietanes with differing double bond configurations (levopimaradiene, abietadiene, neoabietadiene and palustradiene (31)). Minor amounts of dehydroabietadiene are also frequently present in assays with TPS-LAS enzymes. All panels; peak and chemical structure numbers correspond to compounds 1 through 5 in Fig. 1, and

numbering continues for additional terpene synthase products as follows: abietadiene (6), neoabietadiene (7), palustradiene (8), dehydroabietadiene (9). (*D*) Selected ion monitoring (SIM) chromatograms of the 10 ancestral enzymes and PaTPS-LAS when assayed with GGPP or CPP. Diagnostic ion *m/z* = 247 $[M – C_3H_7]^+$ was monitored to determine which mixed product profiles resulted from degradation of 13-hydroxy-8(14)-abietene (31). Traces for no-substrate controls are shown in gray in all relevant panels.

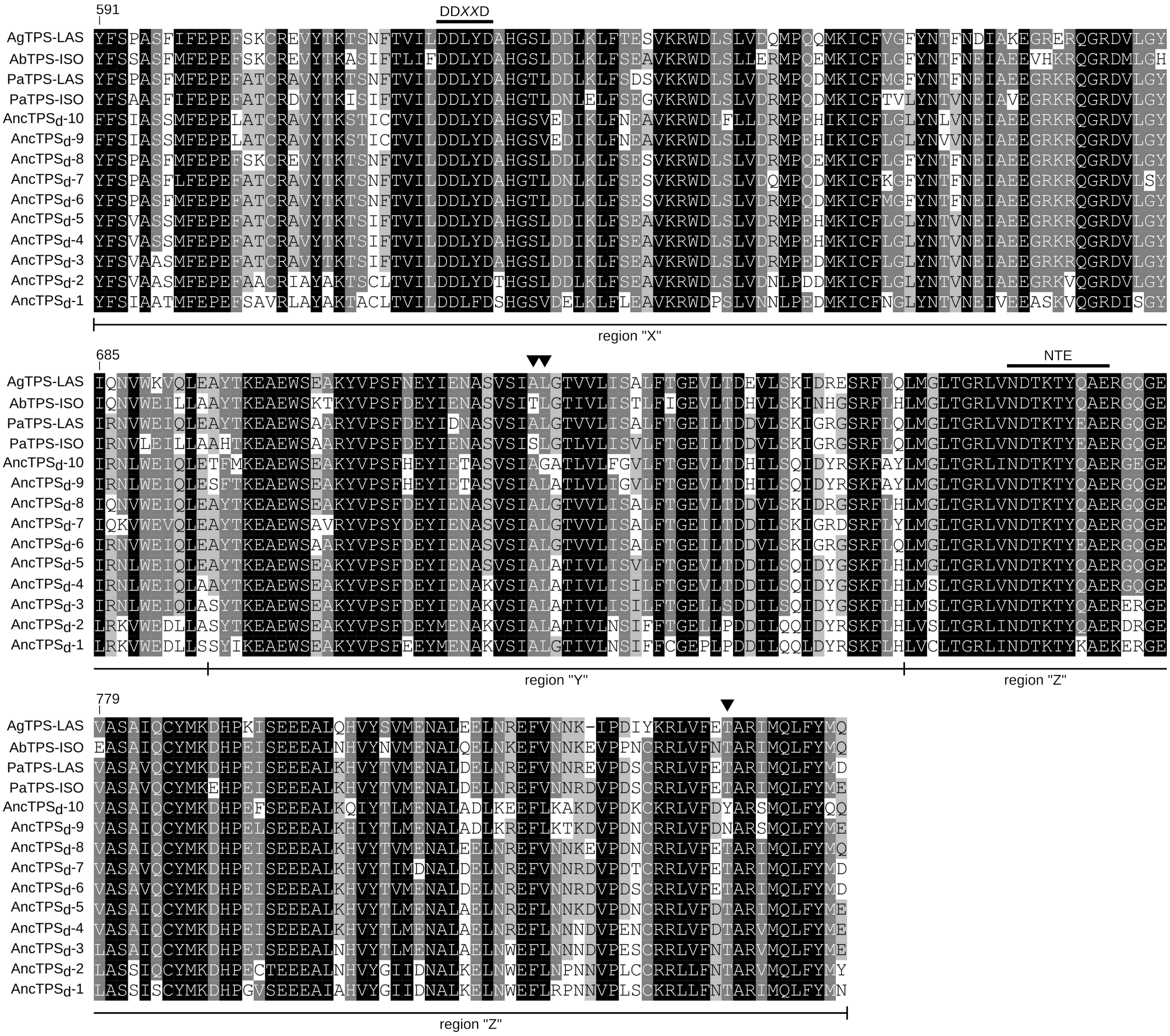

Fig. S6. Amino acid alignment of regions “X”, “Y” and “Z” of ancestral TPS-d3 enzymes and selected extant enzymes. The three protein regions as defined here span a major subset of the “alpha” domain, from amino acid residue 591 through residue 843 as numbered according to the published *Abies grandis* TPS-LAS (AgTPS-LAS, cited in the main text). Each region is indicated below the alignment. Residues that affected catalytic function are indicated by black triangles. “DD*XX*D” and “NTE” motifs are both required for substrate positioning and catalysis and are indicated by horizontal lines above the alignment. “Ag”, *A. grandis*; “Ab”, *A. balsamea*; “Pa”, *Picea abies*.

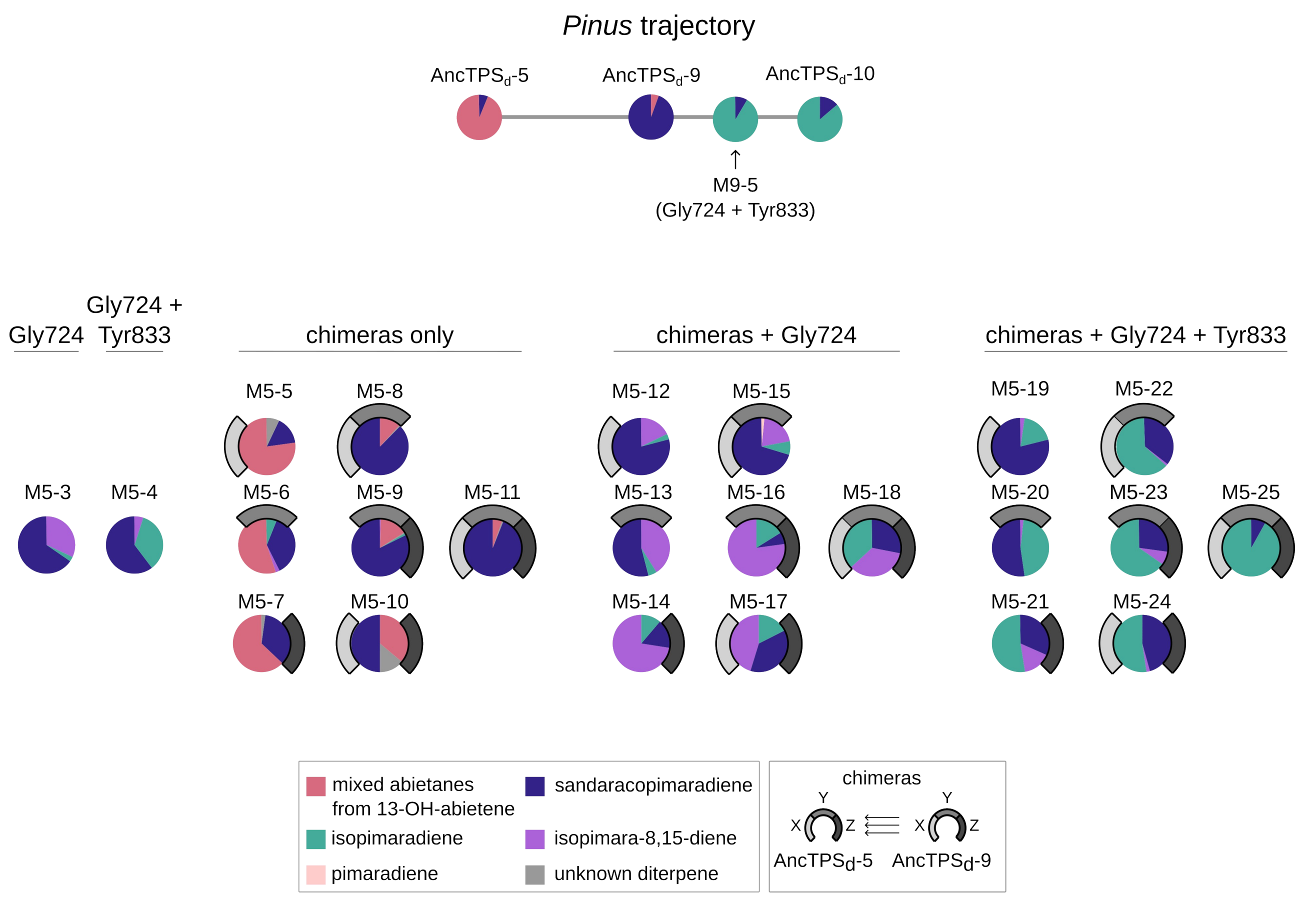

Fig. S7. The effects of lineage history between AncTPS$_d$-5 and AncTPS$_d$-9 on replacements with Gly724 and Tyr833. A schematic of historically realized replacements in *Pinus* is shown at the top of the figure. AncTPS$_d$-5 was converted to an enzyme that promotes isopimaradiene formation through replacements with Gly724 and Tyr833 by introducing regions "X", "Y", and "Z" (Supplementary Fig. 6) from AncTPS$_d$-9 in all possible combinations. Color scheme for all panels and chimera configurations are shown in the boxed legends at the bottom of the figure. Mixtures of abietanes produced by mutants of TPS-LAS-like enzymes are assumed to be due to residual formation of 13-hydroxy-8(14)-abietene (13-OH-abietene).

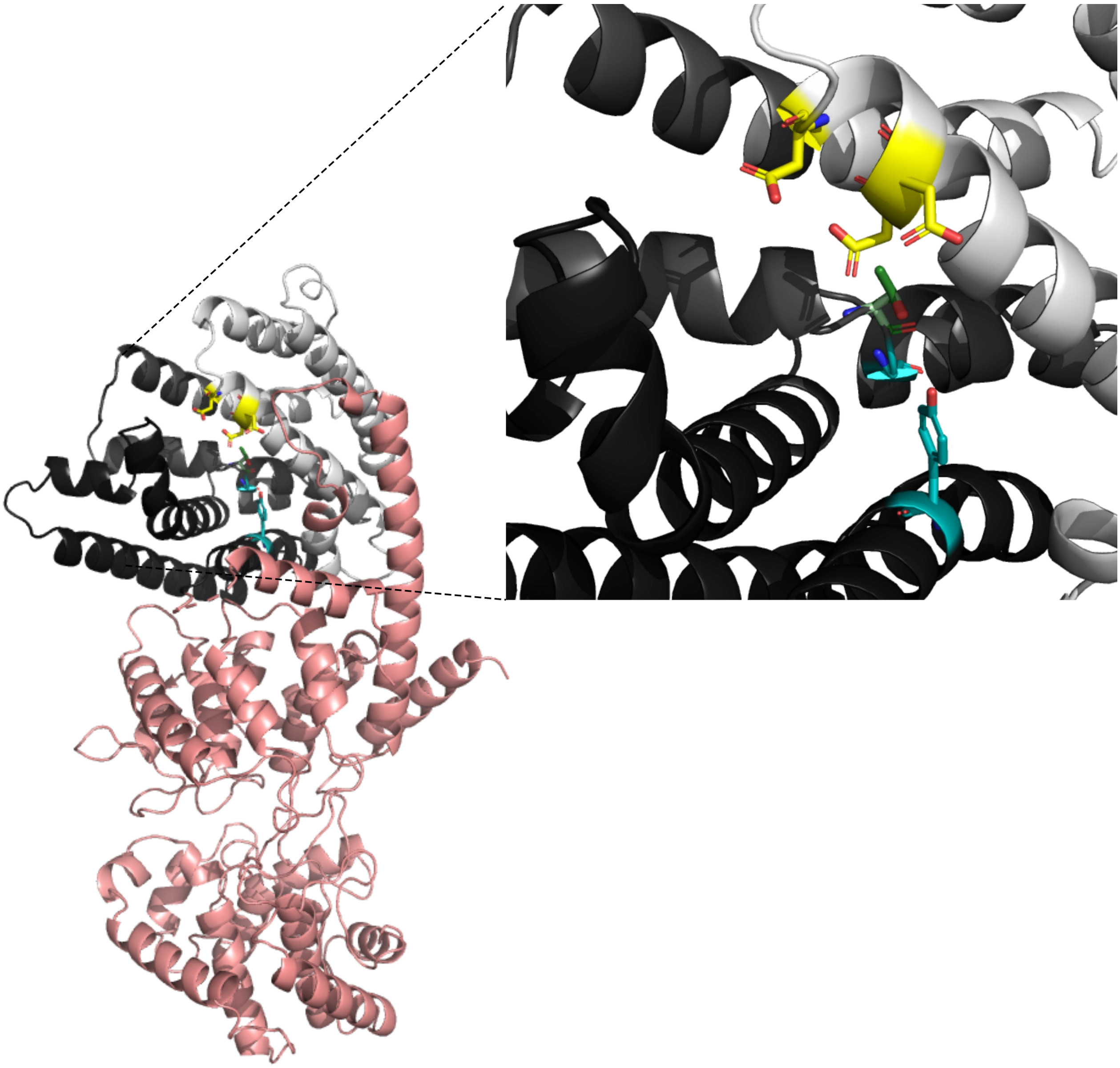

Fig. S8. Active site positions of derived residues following site-directed mutagenesis, chimeric regions, and the conserved DD*XX*D motif. The published structure of *Abies grandis* TPS-LAS (PDB 3S9V) (32) is shown in the lower-left. Regions “X”, “Y”, and “Z” in the protein alpha domain, as cited in the main text, are shown in light gray, gray and black, respectively. The A723S and A723T mutations result in derived residues that are superimposed and are shown in light green and green, respectively. The derived residues following L724G and N833Y replacements are shown in teal. Aspartic acid residues of the DD*XX*D motif required for class-I catalysis are shown in yellow. The blow-out in the upper-right shows the binding pocket for the class-I substrate and the site of class-I catalysis (32).

SI References

1. J. H. Leebens-Mack et al., One thousand plant transcriptomes and the phylogenomics of green plants. *Nature* **574**, 679-685 (2019).
2. C. W. DePamphilis et al., The *Amborella* genome and the evolution of flowering plants. *Science* **342***,* 1241089 (2013).
3. B. Lu, T. Shi, J. Chen, Chromosome-level genome assembly of watershield (*Brasenia schreberi*). *Sci Data* **10**, 467 (2023).
4. L. Zhang et al., The water lily genome and the early evolution of flowering plants. *Nature* **577**, 79-84 (2020).
5. J. Ma et al., The *Chloranthus sessilifolius* genome provides insight into early diversification of angiosperms. *Nat Commun* **12**, 6929 (2021).
6. L. Qin et al., Insights into angiosperm evolution, floral development and chemical biosynthesis from the *Aristolochia fimbriata* genome. *Nat Plants* **7**, 1239-1253 (2021).
7. S. M. Chaw et al., Stout camphor tree genome fills gaps in understanding of flowering plant genome evolution. *Nat Plants* **5**, 63-73 (2019).
8. C. Y. Cheng, V. Krishnakumar, A. P. Chan, F. Thibaud-Nissen, S. Schobel, C. D. Town, Araport11: a complete reannotation of the *Arabidopsis thaliana* reference genome. *Plant J* **89**, 789-804 (2017).
9. E. Mosca et al., A reference genome sequence for the European silver fir (*Abies alb*a Mill.): A community-generated genomic resource. *G3 Genes, Genomes, Genet* **9**, 2039-2049 (2019).
10. Y. Liu et al., The *Cycas* genome and the early evolution of seed plants. *Nat Plants* **8**, 389-401 (2022).
11. R. Guan et al., Draft genome of the living fossil *Ginkgo biloba*. *Gigascience* **5**, 49 (2016).
12. B. Nystedt et al., The Norway spruce genome sequence and conifer genome evolution. *Nature* **497**, 579-584 (2013).
13. A. Zimin et al., Sequencing and assembly of the 22-Gb loblolly pine genome. *Genetics* **196**, 875-890 (2014).
14. H. Lou et al., The *Torreya grandis* genome illuminates the origin and evolution of gymnosperm-specific sciadonic acid biosynthesis. *Nat Commun* **14**, 1315 (2023).
15. T. Wan et al., The *Welwitschia* genome reveals a unique biology underpinning extreme longevity in deserts. *Nat Commun* **12**, 4247 (2021).
16. J. Zhang et al., The hornwort genome and early land plant evolution. *Nat Plants* **6**, 107-118 (2020).
17. J. L. Bowman et al., Insights into land plant evolution garnered from the *Marchantia polymorpha* genome. *Cell* **171**, 287-304 (2017).
18. D. Lang et al., The *Physcomitrella patens* chromosome-scale assembly reveals moss genome structure and evolution. *Plant J* **93**, 515-533 (2018).
19. J. A. Banks et al., The Selaginella genome identifies genetic changes associated with the evolution of vascular plants. *Science* **332**, 960-963 (2011).
20. F. W. Li et al., Fern genomes elucidate land plant evolution and cyanobacterial symbioses. *Nat Plants* **4**, 460-472 (2018).
21. D. B. Marchant et al., Dynamic genome evolution in a model fern. *Nat Plants* **8**, 1038-1051 (2022).
22. D. M. Martin, J. Fäldt, J. Bohlmann, Functional characterization of nine Norway spruce TPS genes and evolution of gymnosperm terpene synthases of the TPS-d subfamily. *Plant Physiol* **135**, 1908-1927 (2004).
23. K. Katoh, D. M. Standley, MAFFT multiple sequence alignment software version 7: Improvements in performance and usability. *Mol Biol Evol* **30**, 772-780 (2013).
24. A. M. Kozlov, D. Darriba, T. Flouri, B. Morel, A. Stamatakis, RAxML-NG: A fast, scalable and user-friendly tool for maximum likelihood phylogenetic inference. *Bioinformatics* **35**, 4453-4455 (2019).
25. K. Hayashi, H. Kawaide, M. Notomi, Y. Sakigi, A. Matsuo, H. Nozaki, Identification and functional analysis of bifunctional *ent*-kaurene synthase from the moss *Physcomitrella patens*. *FEBS Lett* **580**, 6175-6181 (2006).
26. G. Yu, D. K. Smith, H. Zhu, Y. Guan, T.T.-Y. Lam, ggtree: an R package for visualization and annotation of phylogenetic trees with their covariates and other associated data. *Methods Ecol Evol* **8**, 28-36 (2017).
27. R. J. Peters, M. M. Ravn, R. M. Coates, R. B. Croteau, Bifunctional abietadiene synthase: Free diffusive transfer of the (+)-copalyl diphosphate intermediate between two distinct active sites. *J. Am. Chem. Soc.* **123**, 8974-8978 (2001).
28. L.-T. Ma et al., Biochemical characterization of diterpene synthases of *Taiwania cryptomerioides* expands the known functional space of specialized diterpene metabolism in gymnosperms. *Plant J.* **100**, 1254-1272 (2019).
29. Y. Mao et al., Discovery of class I diterpene cyclases producing a tetracyclic cephalotene skeleton in Plum Yews. *ACS Catal.* **13**, 8600-8612 (2023).
30. Z. Yang, PAML 4: Phylogenetic analysis by maximum likelihood. *Mol. Biol. Evol.* **24**, 1586-1591 (2007).
31. C. I. Keeling, L. L. Madilao, P. Zerbe, H. K. Dullat, J. Bohlmann, The primary diterpene synthase products of *Picea abies* levopimaradiene/abietadiene synthase (PaLAS) are epimers of a thermally unstable diterpenol. *J. Biol. Chem.* **286**, 21145–21153 (2011).
32. K. Zhou et al., Insights into diterpene cyclization from structure of bifunctional abietadiene synthase from *Abies grandis*. *J. Biol. Chem.* **287** 6840–6850 (2012).
